# Alteration of Sulfur-Bearing Silicate-Phosphate (Agri)Glasses in Soil Environment: Structural Characterization and Chemical Reactivity of Fertilizer Glasses: Insights from ‘In Vitro’ Studies

**DOI:** 10.3390/molecules30081684

**Published:** 2025-04-09

**Authors:** Anna Berezicka, Justyna Sułowska, Magdalena Szumera

**Affiliations:** Faculty of Materials Science and Ceramics, AGH University of Krakow, Al. A. Mickiewicza 30, 30-059 Krakow, Poland; sulowska@agh.edu.pl

**Keywords:** slow-release fertilizers, sulfate-bearing glasses, nutrient leaching, glass structure, dissolution kinetics

## Abstract

Vitreous carriers of essential nutrients should release elements in response to plant demand, minimizing over-fertilization risks. This study focused on designing and characterizing sulfate-bearing slow-release fertilizers based on four glass series (41SiO_2_∙6(10)P_2_O_5_∙20K_2_O–33(29)MgO/CaO/MgO + CaO) with increasing sulfate content. Structural analysis identified a network dominated by $Q_{\mathrm{Si}}^{2}$ units, with some $Q_{\mathrm{Si}}^{3}$ species and isolated $Q_{P}^{0}$ units. This fragmented structure resulted in high solubility in acidic environments while maintaining water resistance. Such dual behavior is a direct consequence of the delicate balance between depolymerized silicate chains and isolated orthophosphate units, which ensure rapid ion exchange under acidic conditions while preventing uncontrolled leaching in neutral media. Nutrient leaching depended on SO_3_ content, affecting matrix rigidity, and on the type of alkaline earth modifier and P_2_O_5_ content. Dissolution kinetics showed an initial rapid release phase, followed by stabilization governed by silicate hydrolysis. Thermal analysis linked network flexibility to dissolution behavior—CaO promoted an open structure with high SiO_2_ release, MgO increased rigidity, while their co-addition reduced ion diffusion and silica dissolution. The thermal behavior of the glasses provided indirect insight into their structural rigidity, revealing how compositional variations influence the mechanical stability of the network. This structural rigidity, inferred from glass transition and crystallization phenomena, was found to correlate with the selective dissolution profiles observed in acidic versus neutral environments. These results reveal complex interactions between composition, structure, and nutrient release, shaping the agricultural potential of these glasses.

## 1. Introduction

The concept of glass fertilizers originated from the observation of natural processes and an effort to replicate them. Volcanic activity, despite its destructive impact, has given rise to some of the most fertile soils on Earth, which owe their unique properties to the presence of volcanic glass—one of the first components to undergo weathering. As glass decomposes, essential cations are gradually released into the soil, making them bioavailable for plant uptake [1,2,3].

While mineral weathering plays a crucial role in sustaining long-term soil fertility, it alone may not provide a sufficient supply of nutrients to support optimal plant growth or soil revitalization. Remarkedly, soil is far from being an inert medium; it is a complex, living ecosystem teeming with microorganisms that work in symbiosis to maintain nutrient availability. Both plants as well as rhizosphere bacteria and fungi contribute to this process and respond to nutrient deficiencies by exuding organic acids, sugars, vitamins, inorganic ions, and enzymes, among other compounds, to mobilize nutrients from soil minerals, further enhancing nutrient cycling [4,5,6].

A well-designed glass fertilizer integrates seamlessly into this dynamic system, responding to the biochemical signals of the rhizosphere while preserving the delicate biogeochemical balance essential for sustainable soil health [3,7,8,9,10,11,12,13].

Achieving this ‘proper design’ is critical to ensuring the final product exhibits the desired properties. However, despite the apparent simplicity of the glass fertilizer concept, its fabrication presents significant challenges. The final material must meet several key requirements:

- Targeted composition—the glass must incorporate specific macro- and micronutrients to address existing soil deficiencies effectively;
- Amorphous structure—the material should exist in a fully vitreous state to enhance its solubility and reactivity compared to its crystalline counterparts;
- Selective solubility—rather than dissolving readily in water, the glass should respond to acidic exudates from plant roots and soil microorganisms, ensuring nutrients are released only when needed;
- Slow release—nutrient leaching should occur gradually throughout the entire plant development cycle, providing a sustained supply of essential elements.

Regarding the first of the aforementioned requirements, extensive research has shown that a silicon-oxygen matrix provides a highly adaptable framework capable of accommodating a wide range of beneficial ions [3,14,15,16,17,18,19]. These include not only well-established macronutrients such as PO_4_^3−^, K^+^, Mg^2+^, or Ca^2+^, but also less conventional ones, such as SO_4_^2−^, recently acknowledged as the fourth major nutrient element, alongside NPK—a designation well justified by its critical role in protein structure and synthesis, enzyme and vitamin function, and plant defense mechanisms against stress and pests [20,21,22,23,24,25,26,27,28,29,30].

While achieving the first requirement may seem straightforward, it is inherently linked to the second—the selected components must not only be beneficial for the intended application but also capable of forming a stable vitreous network. Although it is commonly believed that any material can be transformed into a glass if cooled rapidly enough from the molten state to prevent nucleation and crystal growth, obtaining fully amorphous materials is often far more complex [31,32,33,34,35,36]. This difficulty arises from the fact that a glassy state represents a departure from the thermodynamically favored, long-range periodic lattice arrangement. Hence, even if a liquid is successfully frozen into a glass by restricting atomic mobility and preventing rearrangement into equilibrium lattice sites, the system will gradually and imperceptibly shift toward its energy minimum over time [37,38,39,40,41]. This challenge becomes particularly pronounced in multicomponent systems, where elements with substantial differences in bonding nature and chemical properties must coexist within a common network. Such structural incompatibilities introduce internal stresses that lower the energy barrier for nucleation, increasing the likelihood of crystallization. This issue is especially relevant for SiO_2_–P_2_O_5_-based glass fertilizers and sulfate-containing oxide glass systems, both of which exhibit a strong tendency toward phase separation or crystallization [42,43,44]. The requirement of obtaining fully amorphous carriers of essential nutrients is not arbitrary. The glassy state, being a higher-energy configuration, is significantly more soluble than its crystalline counterpart, in which the presence of long-range order and the absence of strained, reactive Si–O–Si bonds confer superior chemical durability by limiting hydrolysis sites and restricting water penetration into the bulk [45,46,47,48,49,50]. Thus, ensuring an amorphous structure is critical to enhancing the controlled release of useful ions, making glass fertilizers’ behavior effective and predictable in their intended applications.

Careful selection of chemical composition is also crucial for meeting the third and fourth prerequisites. Unlike crystalline soil minerals, which weather too slowly to support plant growth, or conventional mineral fertilizers, which release nutrients rapidly and hence are prone to leaching losses, glass fertilizers can strike a balance—offering a controlled, responsive supply of essential elements. In the case of conventional mineral fertilizers, even regular rainfall can cause excessive and uncontrolled nutrient release from their chemical structure, leading to losses and potential environmental pollution. This release is dictated by plant-driven biochemical signals, primarily the exudation of weak organic acids, ensuring nutrients become available precisely when they are needed [51,52,53,54,55,56].

The sustained nutrient release from glass fertilizers is driven by processes occurring in their near-surface regions under the influence of chemically active soil components. The release rate is primarily governed by the concentration of exogenous protonated species (H^+^ or H_3_O^+^) in the surrounding environment as well as the structural characteristics of the glass matrix. Specifically, the degree of network connectivity and its openness dictate the susceptibility of Si–O–Si linkages to hydrolysis and water infiltration, respectively, both controlling the pace of dissolution and the availability of nutrients over time [57,58,59].

Remarkedly, such chemical activity of glass fertilizers is inherently tied to their structural characteristics, which, in turn, are dictated by material’s chemical composition—emphasizing the fundamental composition–structure–property relationship. Since glass chemical activity is founded on the gradual release of its constituents into the soil solution, it is significantly influenced by the degree of network cross-linking and the strength of the bonds between network formers and modifier cations [60,61]. Consequently, the transition from highly soil-reactive to chemically inert glass compositions can primarily be controlled by adjusting the content of network-forming oxides, particularly SiO_2_ and P_2_O_5_ [62].

As demonstrated by Edèn [63], materials with an ‘disconnected’ network—composed predominantly of disrupted silicate chains (primarily $Q_{\mathrm{Si}}^{2}$ tetrahedra, with minor $Q_{\mathrm{Si}}^{3}$ and $Q_{\mathrm{Si}}^{1}$ contributions)—exhibit enhanced interaction with biological solutions compared to three-dimensional networks dominated by $Q_{\mathrm{Si}}^{3}$ and $Q_{\mathrm{Si}}^{4}$ species. It is worth emphasizing that in the Q^n^ notation, used to describe the structure of silicate (or phosphate) glasses, Q stands for ‘quaternary’, while n indicates the number of bridging oxygens (BO, bridging oxygens) in a given SiO_4_ or PO_4_ polyhedron (meaning that the remaining 4−n are non-bridging oxygens, NBO). Since a bridging oxygen (BO) links two adjacent tetrahedra, these factors reflect the overall network connectivity (NC) of the glass: n = 2 corresponds to infinite chains or rings, n = 3 represents sheet-like structures, and n = 4 refers to a three-dimensional network framework. Furthermore, compositions exhibiting the strongest response to biological fluids typically have a network connectivity (NC) of approximately 2–3 [60]—such a loosely connected structure facilitates the surface penetration of physiological solutions into the glass, granting access to modifier ions embedded within the matrix, thereby distinguishing bioactive (or soil-active) glasses from their conventional counterparts [64].

The influence of P_2_O_5_ content on glass structure and, consequently, its chemical activity is more complex than that of silicon. Generally, when phosphorus is primarily present as isolated $Q_{P}^{0}$ species, charge-balanced by modifier cations, and the silicate subnetwork maintains an NC within the chemically active range (≈2–3), an increase in its content enhances glass’s interaction with biological solutions [63]. This is attributed to the facile release of isolated PO_4_^3−^ groups, which concurrently accelerate the formation of a metastable layer of slow dissolution on the glass surface—an essential factor in regulating the slow-release properties of glass fertilizers [60,62]. However, excessive phosphorus concentrations can deplete the reservoir of available modifier cations, leading to the clustering of orthophosphate tetrahedra and the emergence of $Q_{P}^{1}$ and $Q_{P}^{2}$ species. This structural shift significantly reduces glass solubility and reactivity in biological environments, as demonstrated by Edèn [63] and further corroborated by Ting et al. [65], who observed that the presence of polyphosphate chains markedly increased resistance to physiological fluids. Additionally, mixed-network glasses with high P_2_O_5_ and low SiO_2_ content exhibit improved mechanical and thermo-mechanical properties, which can be fine-tuned by adjusting silica levels in the composition [66,67].

An alternative approach to modifying the glass structure—and consequently its susceptibility to degradation in biological environments—involves adjusting the proportion of glass components that alter the vitreous network by converting bridging oxygens (BOs), which ionically-covalently link adjacent polyhedra, into non-bridging oxygens (NBOs). This transformation results in the formation of Si(P)-O-M^+^ linkages, where M⁺ represents a modifier cation. The incorporation of increasing amounts of such network constituents, typically alkali or alkaline earth metal oxides, into an initially fully polymerized silicate glass structure, leads to a reduction in network connectivity, thereby enhancing the material’s susceptibility to dissolution [62,64,68].

Equally crucial for the intended application is the nature of the bonds between modifier cations and the glass network components. The predominantly ionic character of Si(P)-O-M^+^ linkages weakens the overall structural integrity and chemical resistance of the glass, making these elements the first to be released upon exposure to a corrosive medium [14,15,43,62,69]. Interestingly, despite their analogous role in modifying the vitreous matrix, different cations can induce significant structural variations. Factors such as ionic radius, field strength, oxidation state, coordination number, and bonding type with the glass network collectively influence material properties, including connectivity, density, hardness, thermal behavior, framework rigidity, and stability [59,70,71]. For instance, substituting Mg^2+^ with the larger Ca^2+^ expands the silicon-oxygen backbone, increasing its permeability to protonated exogenous species, thereby facilitating ion exchange and dissolution [59]. Furthermore, when a glass contains a mixture of alkali and/or alkaline earth ions, it may exhibit nonlinear behavior relative to compositions containing only a single type of modifier ion—a phenomenon known as the mixed-alkali or mixed-alkaline earth effect [70,71,72]. In such cases, ion diffusion may be hindered due to the presence of modifier cations of different sizes occupying non-interchangeable sites—coordination environments within the glass matrix that are structurally and energetically optimized for specific cation types and thus cannot be effectively occupied by different cations due to mismatched ionic radii, charge densities, or bonding preferences—which ultimately disrupts ion migration pathways and suppresses ionic mobility, a hallmark of the mixed-alkaline earth effect, thereby affecting the chemical activity of the glass [70].

While the effects of the above-mentioned network components on the chemical activity of silicate-phosphate glasses—though highly dependent on composition and concentration—are relatively well understood, the influence of sulfur remains an open question. Due to the poor miscibility between silica-rich and sulfur-rich liquids under low-pressure conditions [73], incorporating [SO_4_^2−^] species into the vitreous network presents a considerable challenge. Moreover, to the best of the authors’ knowledge, no studies have yet established a direct link between the composition-dependent structure of sulfate-bearing silicate-phosphate glasses and their chemical activity.

Thus, the primary objective of this study is to elucidate the intricate relationships between glass composition, structural evolution, and chemical reactivity in sulfate-containing silicate-phosphate systems. The following sections present a preliminary investigation of various glass formulations, an analysis of their structural transformations, and an assessment of their chemical activity in relevant solutions. These findings aim to provide essential guidelines for the rational design and characterization of materials intended for environmental protection.

## 2. Results and Discussion

### 2.1. Preliminary Glass Investigation

Prior to evaluating the potential of the designed glasses as slow-release vitreous fertilizers, a preliminary analysis was conducted to comprehensively understand the composition–structure–property relationships within the context of the tested samples.

#### 2.1.1. Assessing the Amorphous Nature of Glasses (XRD)

**Figure 1:**
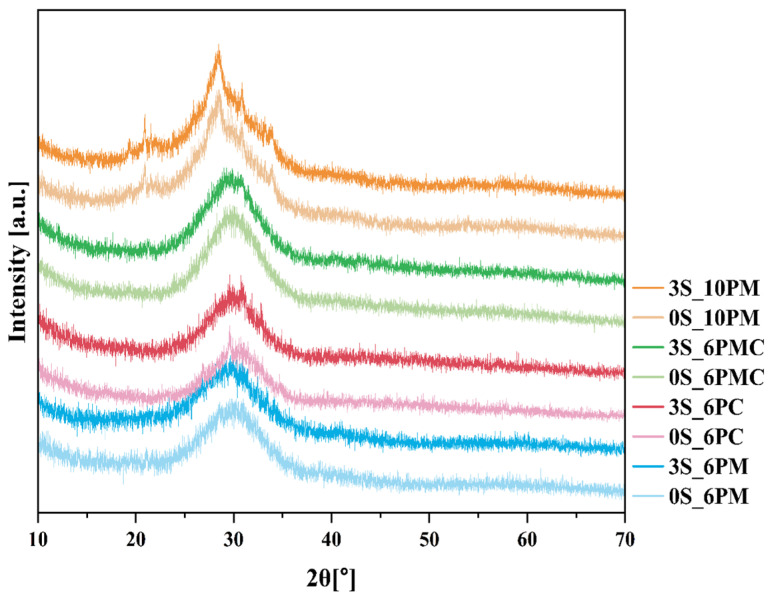
Diffraction patterns of selected samples, including the base composition and those loaded with 3 mol.% sulfate species, from all tested glass formulations.

While induced crystallization is a well-established method for tailoring and controlling the final properties of vitreous materials, spontaneous crystallization constitutes a highly undesirable phenomenon in glasses intended for interaction with biotic systems [50]. A high content of biologically relevant ions (e.g., macro- and micronutrients), which simultaneously act as glass network modifiers, weakens and fragments the structure, while the increased mobility of structural units further promotes their ordering into critical-size nuclei. Not only can crystalline phases, being thermodynamically more stable than their amorphous counterparts, significantly reduce the chemical reactivity of the final product, but the uncontrolled nature of spontaneous crystallization also renders the ion release process unreliable and difficult to predict [48,50,59]. Therefore, XRD analysis was performed not only to verify the amorphous nature of the tested samples but also to track changes induced by the incorporation of sulfur-containing species into the system. The resulting diffractograms of selected glasses (both base samples and those loaded with 3 mol.% sulfate species) are presented in Figure 1.

The primary conclusion drawn from the data presented in Figure 1 is that, while all acquired diffractograms exhibit an elevated background in the 2θ range of 20–40°, characteristic of an amorphous nature, the samples representing the enhanced P_2_O_5_ composition (XS_10PM) also display slightly sharper, more defined reflections, superimposed on the registered halo, indicative of the presence of a crystalline phase embedded within the predominant vitreous matrix.

Given that the early stages of multicomponent glass crystallization are typically governed by diffusion-driven rearrangement of the most mobile and/or weakly bonded species in the structure, and considering that P_2_O_5_ in silicate-based melts generally integrates into the matrix as orthophosphate units, the observed tendency toward spontaneous crystallization in the [10P] composition is unsurprising [59,74,75,76]. This is further supported by the fact that the reflections observed in the diffractograms of both the 0S and 3S_10PM samples indicate the presence of the orthophosphate phase, KMgPO_4_ (Ref. 98-005-0926). On the other hand, phosphate regions in the matrices of lower P_2_O_5_ materials are more diluted within the glass volume, thereby limiting nucleation and restricting the formation of crystalline orthophosphates. The only deviation from this trend is observed in the [6P] composition containing CaO, whose diffractogram exhibits a few low-intensity reflections (at approximately 29.65°, 30.95°, and 33.02° 2θ), suggesting the presence of a small fraction of crystallites (Figure 1). Although the number of detected reflections is limited, their low intensity and overlap with the broad amorphous background prevent definitive phase identification and make their attribution debatable. Considering both the observed peak positions and the aforementioned [PO_4_^3−^] mobility, it is plausible that the phase under evaluation corresponds to potassium calcium phosphate, K_4_Ca(PO_4_)_2_ (Ref. 00-046-0629).

To elucidate the greater crystallization propensity of the XS_6PC system compared to the other [6P] compositions, it is important to highlight the stronger depolymerizing effect of CaO relative to MgO, which consequently increases the tendency of the resulting melt to reorganize during cooling. Furthermore, while MgO is known to act as a network modifier, fragmenting the silicate structure, it also exhibits a dual role in the vitreous matrix, as [MgO_4_^2−^] units can contribute to network formation [59,77,78].

Lastly, the effect of sulfate’s addition on the development of crystalline phases in the studied materials must be considered. A thorough analysis of the recorded diffractograms, along with the authors’ previous research on this phenomenon [79,80,81], suggests an increasing tendency for crystallization with a higher incorporation of sulfate units in the glass. However, one cannot see the effect of SO_3_ content on the progressively more intense and sharper reflections in the XRD patterns of the XS_6PC and XS_10PM systems (Figure 1). This effect arises from the incorporation of sulfate groupings into the flexible amorphous matrix, understood as a loosely cross-linked glass network capable of local structural rearrangements under external stimuli, such as compositional changes or thermal fluctuations. The presence of [SO_4_^2^⁻] tetrahedral units, characterized by highly covalent S–O bonds, disrupts the structural compatibility within the predominantly ionic-covalent silicate framework. These covalent linkages introduce localized distortions and generate internal stresses, which cannot be fully relaxed within a rigid network. As a result, the accumulated stress lowers the energy barrier for atomic rearrangement, enhancing the tendency for spontaneous ordering and ultimately facilitating the formation of critical-size nuclei within the glass matrix [42,82].

#### 2.1.2. Characterization of Thermal Properties

Investigating the thermal behavior of glasses intended for application in biotic environments is of utmost importance. From a technological standpoint, it facilitates the selection of appropriate synthesis temperatures to minimize the risk of spontaneous crystallization, a phenomenon known to reduce glass reactivity in contact solutions, while from an application perspective, differential scanning calorimetry (DSC) analysis serves as an invaluable tool for assessing thermal stability—an essential parameter in the preliminary evaluation of glass chemical activity, as both the crystallization tendency and the release of network-forming elements into the surrounding solution primarily depend on those structural components whose bonds are most susceptible to breaking [15,83].

**Figure 2:**
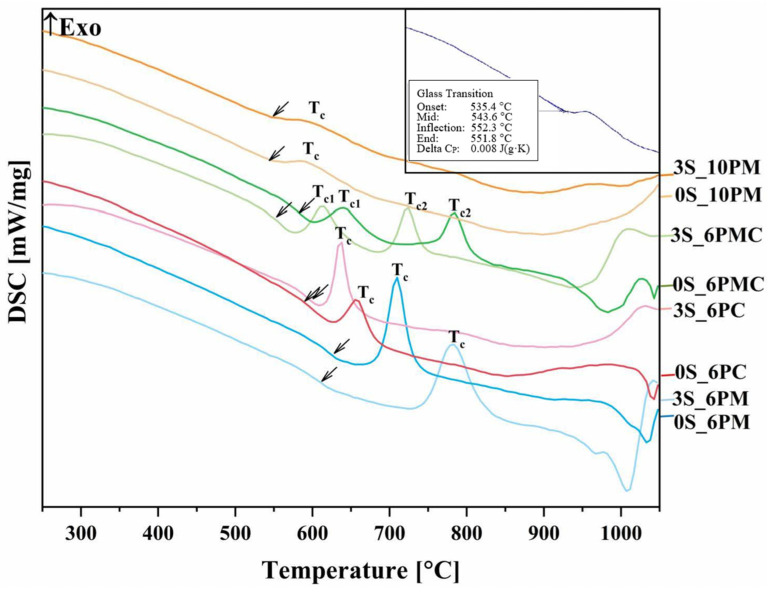
DSC curves of samples from all tested systems: base glasses and those doped with 3 mol.% sulfate. Note: T_g_ values were determined from the inflection points of the DSC signals (right upper corner).

Figure 2 presents the DSC curves, revealing a glass transition followed by a single-stage crystallization event for all glass compositions, except for the system containing both CaO and MgO as alkaline earth modifiers—in this case, two exothermic peaks are observed, reflecting the multicomponent nature of this particular formulation. Notably, while the glass transition and crystallization events in the [6P] series are well-defined, these effects appear significantly less distinct in the [10P] system.

Since the glass transition, manifested as an endothermic baseline shift in the DSC curve, reflects structural relaxation within the vitreous network—its magnitude and temperature being dependent on the nature and concentration of the network-constituting components—the subtlety of this effect may, however, indicate a more rigid internal framework in [10P] glasses. According to Stoch [42,43], glass network rigidity can be influenced by the presence of highly covalent bonds within its structure, but this effect is not straightforward and must be considered in the context of other structural factors. Given this, and considering the complex relationship between [SiO_4_] and [PO_4_] units in terms of polymerization compatibility, the increased P_2_O_5_ content could contribute to internal stresses within the mixed network, potentially leading to some degree of structural destabilization. However, this effect requires further investigation to fully understand its impact.

This, in turn, results in a less pronounced and narrow-range glass transition, characterized by the breaking of strained chemical bonds, followed by the ordering of a random glass structure. Nevertheless, the direct correlation between glass transition behavior and network rigidity remains uncertain. This phenomenon is further reflected in the weak crystallization peak height of the [10P] series observed across all studied systems, rather than the low crystallization temperature itself, which should rather be interpreted as an indicator of a slower crystallization rate (small crystal growth rate) and the difficulty of crystallizing these glasses. This trend is expected due to the higher concentration of glass-forming oxides (SiO_2_ + P_2_O_5_) compared to other formulations, which generally increases the resistance to crystallization.

This phenomenon is further supported by the Angell glass stability parameter (K_A_), which quantifies the material’s propensity for crystallization. The Angell parameter is defined as the difference between the onset of crystallization temperature (T_x_) and the glass transition temperature (T_g_) (K_A_ = T_x_ − T_g_). The term (T_x_ − T_g_) in this equation reflects the material’s resistance to crystallization, where a smaller interval indicates a higher tendency for crystallization (Figure 2, Table 1).

The highest characteristic temperatures together with the highest glass stability parameter are exhibited by the XS_6PM system (Table 1). The lower P_2_O_5_ content, combined with a slightly higher MgO concentration—known for its ability to bond the structure through its capacity to exist in either tetrahedral or octahedral coordination—makes the structure both cohesive and simultaneously more flexible than its higher P_2_O_5_ counterpart. This flexibility allows for structural rearrangement during the glass transition without disrupting the continuity of the network [78,84,85,86].

In contrast to MgO, CaO acts exclusively as a network modifier, leading to relatively lower T_g_ values observed in the XS_6PC system (Table 1). Notably, the extensive fragmentation of Si-O-Si polymeric chains in this modifier-rich system facilitated rapid structural reorganization immediately after the glass transition, which is reflected by the narrow temperature range between T_g_ and T_x_ in the recorded DSC curves of the respective samples and the observed tendency for their spontaneous crystallization, as evidenced by the XRD data presented above (Figure 1).

**Figure 3:**
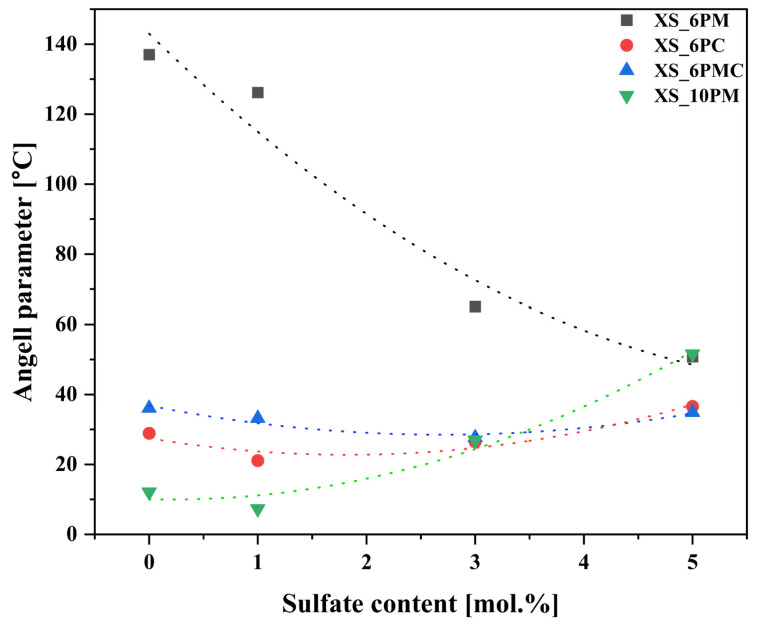
Variation of the Angell parameter across the studied glass compositions. The nominal SO_3_ content is used on the *x*-axis as a reference to distinguish between the samples and does not represent the experimentally measured sulfur concentrations (see Table 3 in Section 4.1).

Meanwhile, introducing both alkaline earth network modifiers into the system placed the XS_6PMC glasses between the XS_6PM and XS_6PC compositions in terms of thermal stability parameters but simultaneously results in lower registered T_g_ values (Table 1). The intermediate K_A_ values relative to the endpoint compositions are understandable and can be directly attributed to the network-forming capabilities of Mg^2+^ as well as its stronger bond to oxygen, which is expected given the higher field strength and smaller ionic radius of magnesium compared to calcium. However, the cause of the more than 50 °C lower T_g_ values for the XS_6PMC system compared to the other [6P] systems remains unclear. Drawing on the work of Kjeldsen et al. [84] and Swansbury et al. [70], it is plausible to hypothesize that this phenomenon may be linked to the mixed alkaline earth effect (MAEE)—the coexistence of Mg^2+^ and Ca^2+^ ions appears to influence the dynamic properties of the glass, potentially impeding the cooperative rearrangement of atoms within the common framework as the system approaches the glass transition, and, subsequently, promoting structural relaxation via bond breakage during the as-accelerated glass transition [42,70,84]. It is important to discuss the impact of sulfate unit addition on the thermal stability of the tested materials, understood as their resistance to devitrification. As shown in Figure 3, the data clearly indicate that the addition of [SO_4_^2−^] species increased the melt’s propensity for ordering its elements and promoting the crystallization of the amorphous material. However, this declining trend continued throughout the entire composition range only for the XS_6PM system, while for the other compositions, a threshold value of sulfate content can be identified, beyond which the trend reversed (an increase in sulfur content leads to enhanced thermal stability). This effect can be attributed to the largest proportion of the amorphous phase in the XS_6PM composition (see Figure 1 as well as the authors’ previous studies on akin materials [79,81])—the absence of a stabilizing crystalline phase led to a gradual weakening of the glass’s internal structure with the introduction of sulfate units, which, by augmenting the number of covalent bonds, progressively increased the rigidity of the internal framework and enhance the system’s tendency for ordering due to bond breaking. While this phenomenon was observed in all studied systems upon the initial addition of [SO_4_^2−^], the increasing network ordering along with sulfur content in the remaining compositions (evidenced by distinct reflections in the XRD patterns for XS_6PC and XS_6PMC systems at 5 mol.% nominal SO_3_ content and for the [10P] system, already in the base sample, see [81] as well as Figure 1) led to an improvement in thermal stability, as reflected by the inversion of the trend (a higher energy barrier for the transformation from a glassy to a crystalline state).

#### 2.1.3. Structural Analysis of Glasses via ^29^Si and ^31^P MAS-NMR Spectroscopy

A key factor that transforms a material traditionally considered inert (e.g., conventional window glass) into one that exhibits chemical activity in biological systems is its fragmented structure. This structural characteristic facilitates the penetration of water molecules into the vitreous network, particularly at sites occupied by network modifiers, thereby enabling the systematic release of ions into the surrounding medium [59,87,88,89].

**Figure 4:**
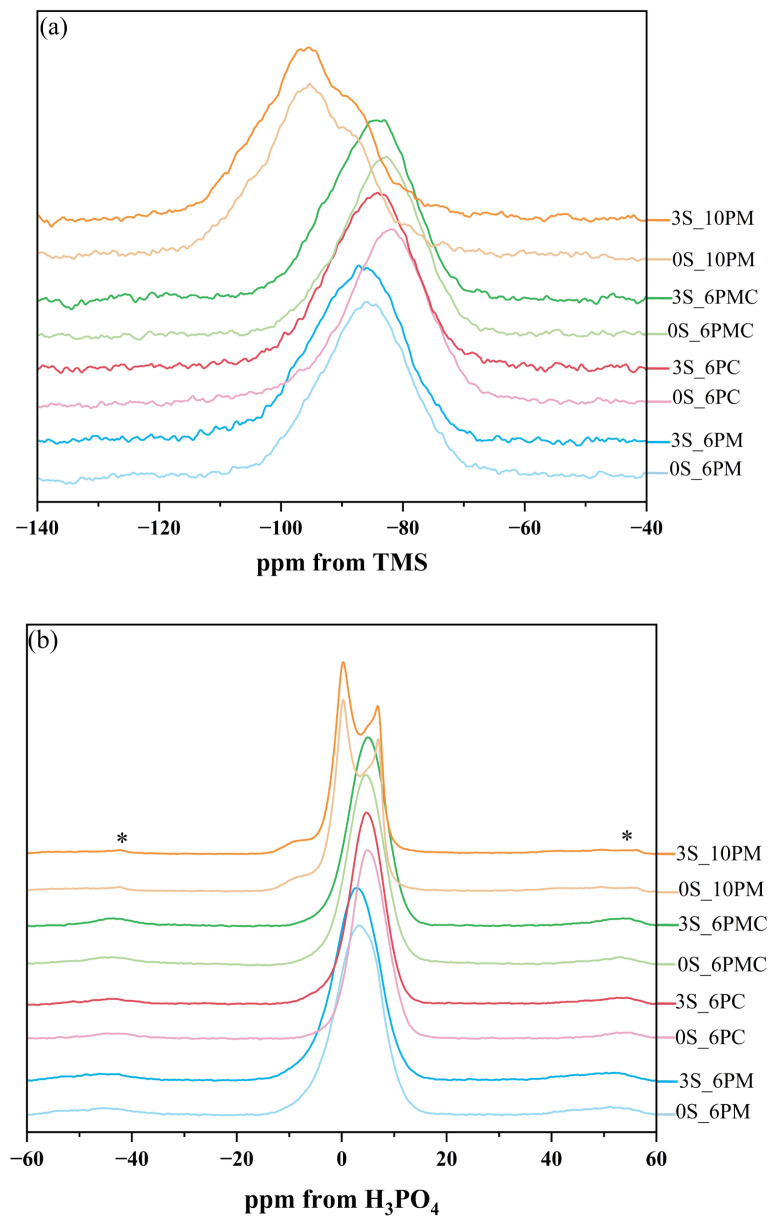
^29^Si (**a**) and ^31^P (**b**) MAS-NMR spectra of the studied glasses (base and those loaded with 3 mol.% sulfate species) for all tested compositions. Spinning sidebands are marked with asterisks.

Therefore, to assess the potential of the designed glasses as vitreous carriers of beneficial ions, ^29^Si and ^31^P MAS-NMR spectroscopy was employed to characterize the vitreous framework of the tested systems. The compilation of MAS-NMR spectra comparing the chemical environments of the network formers in both studied systems ([6P] and [10P]) is presented in Figure 4a (^29^Si MAS-NMR) as well as Figure 4b (^31^P MAS-NMR), respectively.

The obtained ^29^Si MAS-NMR results reveal distinct differences in the distribution of fundamental structural units between the studied compositions, particularly when comparing the [6P] and [10P] series. In glasses with lower P_2_O_5_ content, the positions of the main resonance peaks (ranging from the least negative values: −82.1 ppm for 0S_6PC, −82.9 ppm for 0S_6PMC, and −85.4 ppm for 0S_6PM) indicate a predominantly metasilicate network, primarily composed of $Q_{\mathrm{Si}}^{2}$ species arranged in chains. (The fundamental structural unit of silicate glasses is the SiO_4_ tetrahedron, which can be linked to adjacent SiO_4_ tetrahedra via Si–O–Si bonds, commonly known as bridging oxygens. These structural units are classified as $Q^{n}$ species, where n denotes the number of bridging oxygen atoms attached to a given tetrahedron.) In contrast, the spectrum of the [10P] system exhibits significant asymmetry, suggesting a more chemically diverse structure. While $Q_{\mathrm{Si}}^{2}$ units (−88.7 ppm) remain present, the well-defined resonance at −95.4 ppm signifies the existence of disilicate $Q_{\mathrm{Si}}^{3}$ groups. Additionally, a slight rightward inflection near −78.6 ppm reflects a minor contribution of pyrosilicate $Q_{\mathrm{Si}}^{1}$ units (linked via a single bridging oxygen), while a subtle leftward shoulder at −107.2 ppm indicates the presence of fully polymerized $Q_{\mathrm{Si}}^{4}$ species within the glass network [90,91,92,93,94,95].

Similar trends emerge from the analysis of the ^31^P MAS-NMR spectra (Figure 4b). The signals recorded for glasses with a 6 mol.% P_2_O_5_ content consisted of a single broad resonance, whose position—starting from more positive values—was noted at 4.9 ppm for 0S_6PC and 0S_6PMC and 3.5 ppm for the 0S_6PM system and confirms the exclusive presence of orthophosphate units. Conversely, the resonance observed for the XS_10PM system deviated entirely from this pattern, instead exhibiting two distinct maxima (6.9 and 0.2 ppm), which indicate the existence of two chemically distinct orthophosphate environments in these materials [94,96,97,98].

Beyond providing an initial structural characterization of the synthesized glasses, these results also offer insights into the impact of [SO_4_^2−^] incorporation on the structural evolution of the materials. A comparison of the ^29^Si MAS-NMR resonance positions between the base glasses and those containing 3 mol.% sulfates (shifting to −84.3 ppm for 3S_6PC, −84.11 ppm for 3S_6PMC, −86.5 ppm for 3S_6PM, and −89.3/−95.9 ppm for 3S_10PM) suggests a slight polymerizing effect of [SO_4_^2−^] addition on the silicate framework. A similar, albeit significantly weaker, influence of these groups was observed on the phosphorus-oxygen subnetwork, where a displacement of the main resonance towards more negative values, exceeding 0.5 ppm, was only noted for the 0S_6PM system (3.5 → 2.7 ppm). In the XS_6PC system, the shift was minimal (4.9 → 4.7 ppm), while in the XS_6PMC one, the resonance moved toward more positive ppm values (4.9 → 5.0 ppm). Notably, no alike transition was observed in the XS_10P system.

The obtained results, in line with the general assumption that glasses with a predominantly metasilicate structure tend to interact with biological environments, allow for a preliminary classification of all synthesized glasses as potentially chemically active. Nevertheless, a significantly more reliable measure of such reactivity is the degree of polymerization of the glass network, commonly expressed as network connectivity (NC) [76,99,100]. This parameter, defined as the average number of bridging oxygens (BO) per network-forming element, provides a more quantitative assessment of the structural integrity of the glass framework. The experimental values of network connectivity, ${\text{\{}\mathrm{NC}\text{\}}}_{\exp}$, can be determined based on the exact proportions of individual $Q_{\mathrm{Si}}^{n}$ and $Q_{P}^{n}$ species, following the equations proposed by Eckert, [101], (1)–(3):

(1) $${\left\{NC\right\}}_{Si}=4\times f\left({Si}^{\left(4\right)}\right)+3\times f\left({Si}^{\left(3\right)}\right)+2\times f\left({Si}^{\left(2\right)}\right)+1\times f\left({Si}^{\left(1\right)}\right)$$

(2) $${\left\{NC\right\}}_{P}=3\times f\left(P^{\left(3\right)}\right)+2\times f\left(P^{\left(2\right)}\right)+1\times f\left(P^{\left(1\right)}\right)$$

(3) $${\left\{NC\right\}}_{exp}=\left\{2xP_{2}O_{5}\times {\left\{NC\right\}}_{P}+xS{iO}_{2}\times {\left\{NC\right\}}_{Si}\right\}/x{SiO}_{2}$$

**Figure 5:**
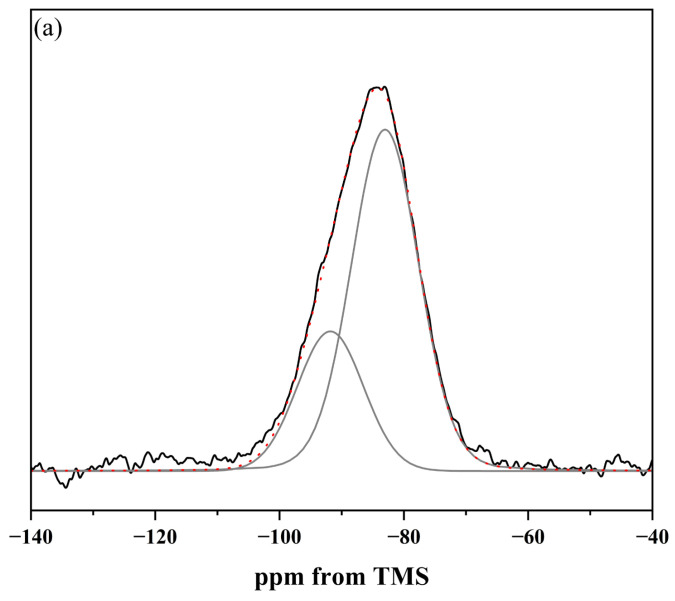

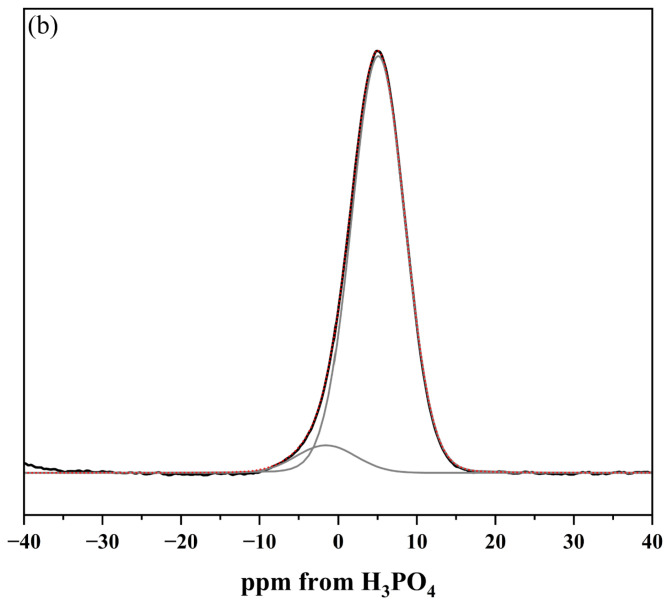
Representative ^29^Si (**a**) and ^31^P (**b**) MAS-NMR spectra of the selected 3S_6PMC sample. The original experimental spectrum is shown in red, while the fitted envelope obtained from spectral deconvolution is marked in black.

**Figure 6:**
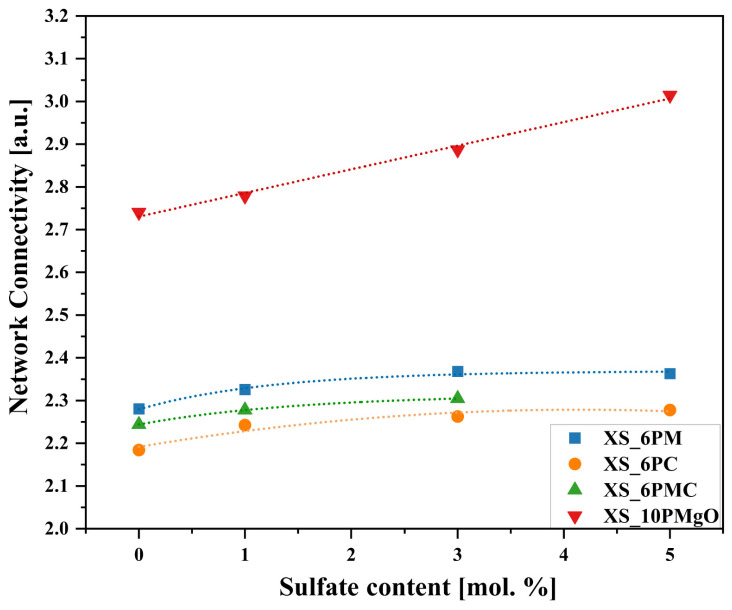
Calculated network connectivity values for the studied glass compositions. The data are plotted against the nominal SO_3_ content, used here solely to distinguish between the samples. Please note that these values do not represent experimentally measured sulfur concentrations, which are provided in Table 3 (see Section 4.1). The NC value for the 5S_6PMC glass is not available due to the specimen’s instability, which prevented the acquisition of both 29Si and 31P MAS NMR spectra.

Therefore, to extract the data necessary for calculations based on Equations (1)–(3) and asses the relative abundance of individual structural units within the glass network as well as the impact of sulfate species’ addition on their distribution, the obtained signals were subjected to a deconvolution procedure. Accordingly, spectral regions were fitted with a set of bands modeled using the Voigt function, which was deemed optimal for approximating most NMR signals. The only exception was the ^31^P MAS-NMR spectra recorded for the [10P] system, where narrow, well-defined resonances were best described by the Lorentzian function. Representative deconvolution results for selected samples from the XS_6PMC system are presented in Figure 5, while a comparison of network connectivity calculations for all glass samples from both studied systems is depicted in Figure 6.

Upon analyzing the obtained network connectivity results, it is evident that the previously stated assumption regarding the higher degree of polymerization in glasses with increased P_2_O_5_ content holds true, as the acquired NC values for XS_10PM samples were significantly higher than those representing the [6P] series (Figure 6). The markedly higher crosslinking observed in the [10P] system should be attributed precisely to its increased P_2_O_5_ content. It is well established that raising the concentration of this network former, without a concurrent increase in modifier oxides necessary for charge balancing the newly formed orthophosphate units, drives the redistribution of M^+^/M^2+^ cations away from the silicate framework and toward [PO_4_^3−^] units. This reorganization of network elements alters the $Q_{\mathrm{Si}}^{n}$ species distribution, ultimately enhancing the connectivity of the silicon-oxygen network [59,102,103]. Additionally, the presence of MgO as the sole alkaline earth network modifier in the [10P] system played a crucial role. As discussed earlier, this particular oxide exhibits characteristics of both a network modifier and former, and thus, a fraction of this component incorporates into the glass matrix as [MgO_4_^2−^] species, which require charge compensation from nearby cations. Given that, similar to [PO_4_^3−^] units, this process effectively increases silicon-oxygen NC; unsurprisingly, the highest Si–O–Si connectivity among all studied [6P] compositions was observed for the XS_6PM system (Figure 6).

Following this reasoning, it becomes evident that CaO exerted a significantly stronger depolymerizing effect on the silicon-oxygen network, which is reflected in the progressive decrease in NC from the XS_6PM system, through the XS_6PMC system (containing an equal ratio of MgO and CaO), to the XS_6PC system (which contains only CaO)—further confirming the sole network-modifying role of calcium oxide (Figure 6). This evident parallel increase in the connectivity of the silicon-oxygen backbone together with an increase in MgO content across the [6P] compositions clearly contradicts the conventional classification of MgO as a network-disrupting component in vitreous systems. Such a statement finds confirmation in the literature data, as according to Jha et al. [86], its role is content-dependent—at concentrations where both MgO and CaO act as network modifiers, differences in glass properties arise from their distinct ionic sizes and field strengths, known to affect the local structure of glasses (e.g., the incorporation of larger cations into the glass structure can enhance the depolymerization of the silicate network [104,105,106]). However, at higher concentrations, MgO begins to form its own network by scavenging modifier cations for charge balancing, thereby reducing the number of non-bridging oxygens in the system. A more detailed perspective is provided by Watts et al. [107], who quantified the network-forming and modifying roles of MgO based on ^29^Si MAS-NMR deconvolution of silicate-based glasses, where MgO was gradually replaced by CaO. Regardless of composition, their findings indicated that while 86% of MgO acts as a traditional network modifier, up to 14% incorporates into the vitreous network as tetrahedral MgO_4_ units, effectively removing modifier cations for charge compensation and promoting network polymerization. Although this behavior may not be universal across all MgO-containing silicate glasses, Watts’ findings suggest that in highly disrupted silicate systems—such as those in her study and the present work—MgO can exhibit a dual, concentration-dependent role. This statement may thus serve as an explanation for the observed decrease in NC as MgO content decreases from XS_6PM through XS_6PMC to its absence in XS_6PC systems.

Despite the above-discussed differences, all the studied systems shared two key similarities—firstly, they all exhibited a similar response to increasing sulfate content, with NC following an upward trend across all materials. The explanation for this behavior mirrors that of the previously discussed [PO_4_^3−^] units: sulfur is introduced into the compositions exclusively as tetrahedral [SO_4_^2−^] species, which attract network modifiers. This redistribution of M^+^/M^2+^ cations disrupts the silicate subnetwork’s continuity, reduces their concentration near the Si-O-Si framework, and favors the formation of more polymerized $Q_{\mathrm{Si}}^{3}$ units.

The second observed similarity, based on the NC trend, is that the values of this parameter for all tested glasses indicate that the silicate network in each case was predominantly formed by $Q_{\mathrm{Si}}^{2}$ units forming silicon-oxygen chains/rings, with a smaller proportion of $Q_{\mathrm{Si}}^{3}$ species constituting the two-dimensional part of the network [59]. This metasilicate-based, open, and fragmented structure places these compositions in the category of glasses that are prone to disintegration in biotic environments. The only system that may raise concerns regarding its potential to interact with aquatic solutions is the [10P] system, where NC exceeds the established threshold for bioactivity (>2.7 [74,99,108]).

Nonetheless, it is important to emphasize here that the network connectivity parameter alone does not capture all the structural aspects that influence glass solubility [59]. The interaction of a vitreous material with a solution is also strongly influenced by the nature of the cross-links (the strength of bonds between modifiers and the silicate-based vitreous matrix) and the P_2_O_5_ content, which, when incorporated into the vitreous matrix as orthophosphate units, directly contribute to the enhanced chemical activity of the glass [3,15,16,74,99,108]. Therefore, to further complete the composition–structure–property relationship and unequivocally assess the potential of the tested compositions as vitreous carriers of useful ions, both ‘in vivo’ and ‘in vitro’ experiments were conducted.

### 2.2. Glass Dissolution Studies (‘In Vitro’ Experiment)

**Figure 7:**
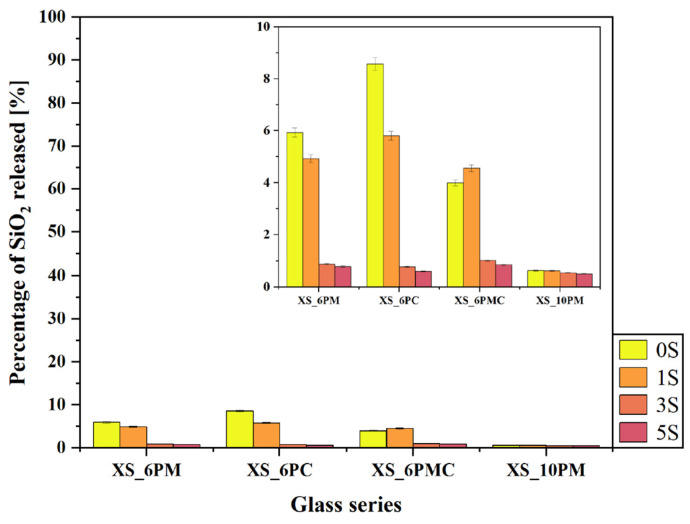
Percentage of SiO_2_ released into the citric acid solution for all studied systems.

**Figure 8:**
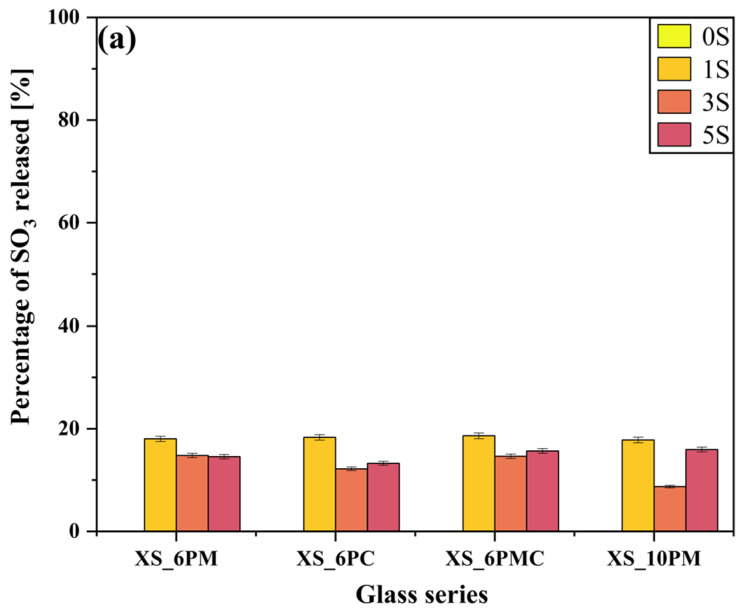

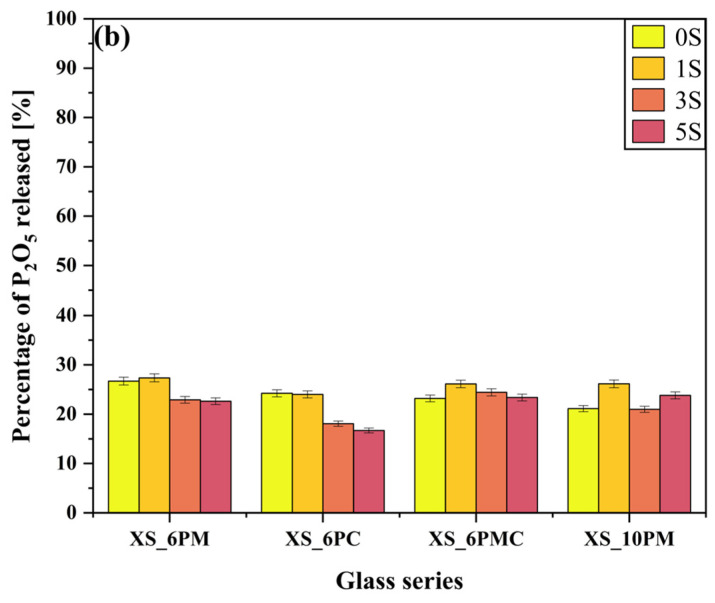
Percentage of SO_3_ (**a**) and P_2_O_5_ (**b**) released into the citric acid solution for all studied systems.

**Figure 9:**
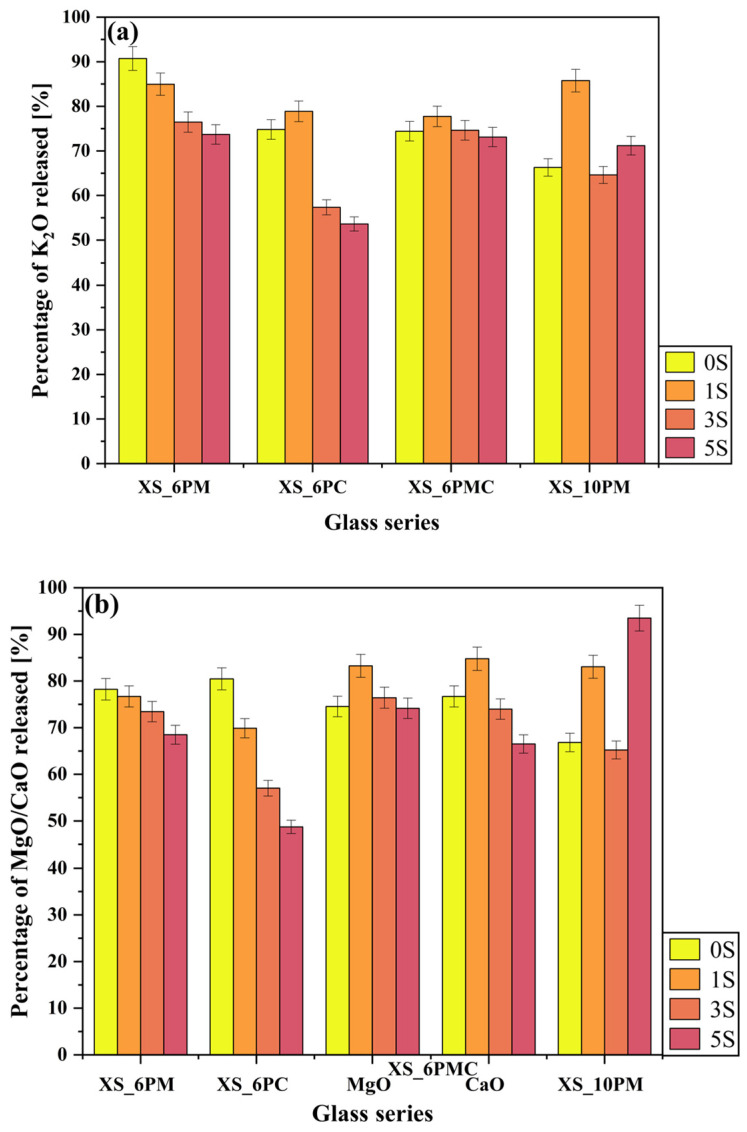
Percentage of K_2_O (**a**) and MgO/CaO (**b**) released into the citric acid solution for all studied systems.

**Figure 10:**
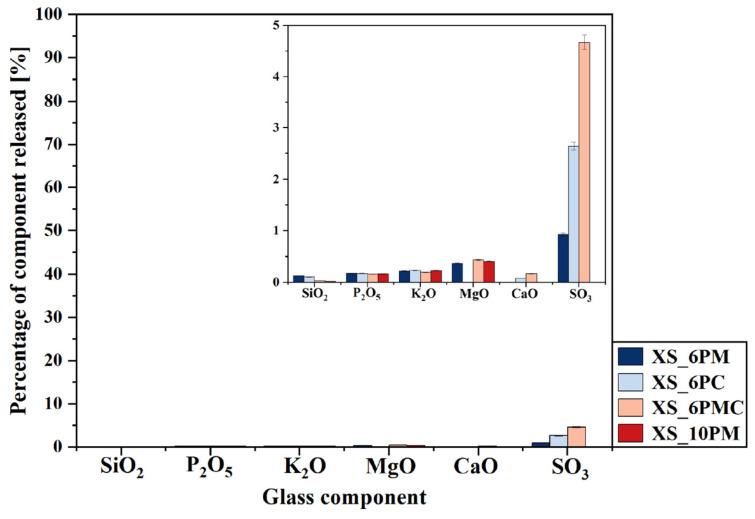
Percentages of glass elements released into distilled water for all studied systems.

**Figure 11:**
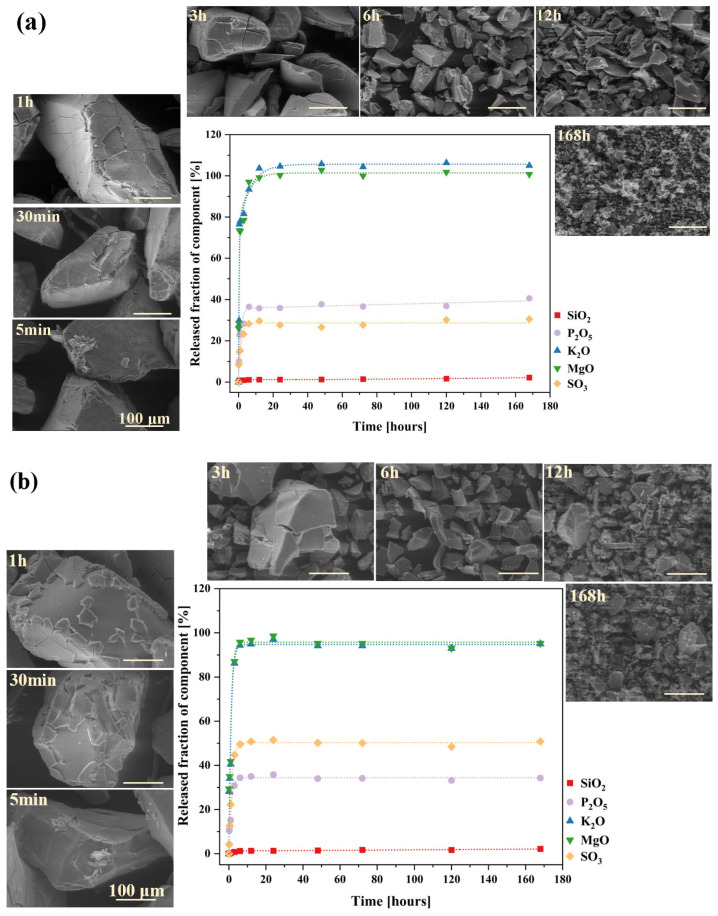
Ion dissolution profiles of 3S_6PM (**a**) and 3S_10PM (**b**) glasses in 2% citric acid under dynamic leaching conditions as a function of immersion time, accompanied by micrographs from selected surface points, illustrating surface changes at various stages of the experiment. It should be stressed that the percentages of K_2_O and P_2_O_5_ released in the case of XS_6PM system were noted to slightly exceed 100% due to the limitations of the XRF analysis (in the pearl method used, the samples were heated to 1100 °C, potentially leading to the volatility of certain glass components and resulting in inaccuracies in the measured values). The micrographs were taken at 1000× magnification, with the scale bar corresponding to 100 µm.

The concept of vitreous fertilizers is based on the controlled release of nutrients in response to plant demand. This interaction between the rhizosphere, soil microorganisms, and the macro-/micronutrient carrier relies on the secretion of low-molecular-weight organic acids, which mobilize elements encapsulated within the glass matrix [52,109,110]. Therefore, to assess the leaching behavior of the designed materials in relation to their chemical composition, an ‘in vitro’ experiment was conducted using a solution that simulates these organic exudates, and the results are presented in Figure 7, Figure 8, Figure 9, Figure 10 and Figure 11.

Importantly, the experiment allowed for assessing the effect of glass composition on sulfate ion release into the surrounding medium. The data presented in Figure 8a indicate that, within the [6P] system, the type of alkaline earth metal oxide modifying the glass matrix had a negligible effect on the release of [SO_4_^2−^] species into the solution, with leached amounts ranging from 10% to 20% of the initial content. Instead, the previously demonstrated network-polymerizing effect induced by sulfate incorporation appears to play a more significant role (see Section 2.1 and [80,81]). Notably, in all systems containing 6 mol.% P_2_O_5_, an increase in [SO_4_^2−^] ion content correlates with a reduced release of this species into the solution. A similar, albeit substantially less pronounced, trend was observed for phosphate ion release (Figure 8b), further supporting the sulfate-induced polymerization effect. However, in this case, the difference between the maximum (~26% for glasses containing ~1 mol.% sulfates) and minimum (~20% for 5S glasses) phosphate release was less significant than the one observed for [SO_4_^2−^]. The release of both anions into the surrounding medium primarily occurs via the hydrolysis of S-O-K^+^ and P-O-K^+^/Mg^2+^/Ca^2+^ bonds, which is strongly promoted by ion exchange in the acidic environment of the citric acid solution [111,112,113]. This process leads to the protonation of phosphate and sulfate units into H_2_PO_4_^−^/HPO_4_^2−^ and HSO_4_^−^, respectively. The sulfate-induced densification of the glass network, therefore, acts as a kinetic barrier, restricting the transport of these relatively large anions to the glass–solution interface. Consequently, the observed leached ion concentrations primarily originate from near-surface regions rather than the glass bulk.

Although the network-polymerizing influence of [SO_4_^2−^] species is also reflected in the release of other glass components of [6P] glasses into the surrounding medium (Figure 7 and Figure 9a,b), it is most pronounced in the data presenting silica release (Figure 7), where a sharp decline in SiO_2_ concentration in the leachate was observed for all glasses beyond the threshold of 1 mol.% SO_3_ (dropping from an average of 6% to just ~1% of the initial content). Numerous scientific reports identify ~1 mol.% SO_3_ as the maximum amount that can be accommodated within the free volume of a silicate-based vitreous framework without triggering crystalline phase precipitation [114,115]. Therefore, a certain correlation can be established between the solubility limits reported in the literature and the data presented in Figure 7. In the systems studied, the precipitation of K_2_SO_4_ as a sulfate-bearing phase was observed at 5 mol.% SO_3_ in both melt-quenched and fritted XS_6PM samples [80], whereas for XS_6PC and XS_6PMC compositions, crystallization occurred at 3 mol.% in melt-quenched glasses, with the fritting process extending this limit to 5 mol.% [79,81].

Beyond the confirmed sulfur-induced polymerization effect, which reduces silica extraction from the glass matrix [80,81], another crucial factor must be then considered—the progressive ordering of the internal glass structure with increasing [SO_4_^2−^] content. Although such structural rearrangements toward a more stable, crystalline-like configuration do not manifest as distinct reflections in the XRD patterns (Figure 1), they undoubtedly enhance the formation of strong, low-strain, hydrolysis-resistant Si–O–Si linkages, ultimately influencing the durability of the glass network [31,42]. This hypothesis is further supported by the stark contrast in dissolution rates between quartz and amorphous silica in pure water, which are 4.2 × 10^−14^ and 9.0 × 10^−13^ mol/m^2^, respectively, underscoring the profound influence of network geometry on dissolution kinetics [45]. Unsurprisingly, the highest silica release was observed for the XS_6PC system, which exhibited the greatest proportion of chain-like $Q_{\mathrm{Si}}^{2}$ units—more susceptible to bond cleavage than the more highly networked $Q_{\mathrm{Si}}^{3}$ units (see Section 2.1.3). As in previous cases, the leached Si most likely originates from near-surface regions, where defect concentrations, including undercoordinated Si atoms, are highest, resulting in increased reactivity [116].

Particular attention should be paid to the release data of the most mobile glass components—potassium ions (K^+^), present in all systems, as well as alkaline earth metal ions (Mg^2+^ and/or Ca^2+^)—in this case, too, the increasing degree of cross-linking in the silicate framework, driven by the rising concentration of [SO_4_^2−^] units, appears to hinder the transport of these species toward the surface (Figure 9a,b). It is well known that the release of cations from the glass network occurs through interactions with positively charged exogenous species, such as H^+^ and H_3_O^+^. Given that alkali metal ions have a lower charge than alkaline earth metal ones, the interdiffusion process typically favors the former [71]. Nonetheless, the investigated glasses within the [6P] series did not follow this general trend, as all network modifier cations were released in comparable amounts (generally between 70–80% of their initial content). The observed lack of preferential leaching of any specific modifier cation can be attributed to the dynamic character of the experiment as well as the MAS-NMR-confirmed open framework of these glasses and the resulting high concentration of hydrophilic non-bridging oxygen atoms, both of which promote deep water penetration [59].

The relationships discussed above were not observed for the glasses representing the [10P] system, with one exception—the silica release profile, where the sulfate-related network-polymerizing effect was again apparent. As the sulfur content in the matrix increased, the release of Si into the solution slightly decreased (Figure 7); however, this effect was rather subtle, as the recorded decline was only from 0.6% to 0.5% of the initial content. In contrast, analysis of the release data for other ions does not reveal a similar influence of sulfate incorporation on their leaching behavior, as was the case for the [6P] series (Figure 7 and Figure 9). This lack of such correlation can be attributed to the increasing instability of the [10P] system with higher sulfate loading, which arises not only from the greater rigidity of the Si-O-Si glass backbone—and consequently, structural stresses—but also from the partial crystallinity of samples representing the higher P_2_O_5_ series, complicating the unequivocal description of ion release data. Undoubtedly then, in these glasses, DSC-confirmed structural instability plays a more dominant role in governing ion release than the presence of silicon-oxygen network-polymerizing sulfate groups.

Analyzing the ‘in vitro’ experimental data, it is worth noting a few cases where an initial increase in the release of a given element (e.g., K^+^ in the case of sample 1S_6PC) was observed compared to the base sample, followed by a subsequent decline. This phenomenon can likely be attributed to a slight expanding effect of [SO_4_^2−^] species on the glass matrix, as these units become localized within its structural voids, thereby facilitating deeper water penetration [59]. However, in later stages, this effect was gradually outweighed by the network-polymerizing influence of sulfate species.

A particularly informative aspect of this ‘in vitro’ study is the analysis of data collected during the exposure of the obtained materials to distilled water (Figure 10), with the most striking observation being the significantly higher release of sulfate ions into the surrounding medium compared to other glass components. Given that none of the remaining structural elements were leached in amounts exceeding 0.5% of their initial content—and that silica was detected in the filtrates at concentrations below 0.1% of its original amount—it can be concluded that the leaching process primarily affected the near-surface regions of the materials. It is noteworthy that the superficial glass area was characterized not only by the presence of weaker bonded atoms and chemically reactive sites (such as non-bridging oxygens, three-coordinated Si, and strained Si-O bonds) but also by a slightly higher concentration of network modifiers compared to the bulk glass [117,118,119].

This assumption leads to the conclusion that the sulfate ions released in such large quantities also originate from the outermost layers of the samples, suggesting at the same time a non-uniform distribution of sulfate units within the glass volume. This interpretation aligns with literature reports describing the tendency of sulfates to form a so-called ‘sulfate gall’ on the surface of silicate melts [114,120,121,122], further supporting the notion of [SO_4_^2−^] species migrating toward the surface [123]. Moreover, the highest sulfate ion concentrations were detected in the filtrates of the 3S_6PC and 3S_6PMC samples, whose melts exhibited the lowest viscosities, potentially facilitating the migration of sulfur species towards the surface. Finally, it is crucial to emphasize the inherently high solubility of sulfate ions in aqueous environments, which, combined with their presence in the glass matrix as isolated units unbound to the network, further enhances the mobility of sulfur species into the surrounding medium [124,125].

Nevertheless, comparing the release of glass matrix components into a corrosive acidic medium (pH~2) versus neutral distilled water clearly demonstrates the high resistance of the investigated materials to the latter. The concentrations of all glass constituents in the water leachate were significantly lower, indicating that the studied materials exhibited enhanced reactivity in environments mimicking root exudates and microbial secretions. These results confirm that the structure of each designed glass composition permitted the penetration of exogenous H^+^ and H_3_O^+^ species into the matrix, facilitating ion exchange and the subsequent liberation of all mobile glass constituents, while silica remained largely retained, with its release not exceeding 10% of the initial content. It is important to emphasize that the ‘in vitro’ experiment, conducted in accordance with Regulation (EC) No 2003/2003 of the European Parliament and of the Council of 13 October 2003 on fertilizers [126], serves as a reliable tool for assessing the leaching potential of specific ions from the tested materials into solutions that simulate those secreted in the rhizosphere. However, as this method does not fully predict or model long-term nutrient leaching, a dissolution kinetics study was conducted to provide deeper insights into release profiles—critical for ensuring a sustained nutrient supply to plants.

### 2.3. Simulation of Long-Term Glass Dissolution (Dissolution Kinetic Studies)

The selection of glass compositions for the study of glass dissolution kinetics was guided by the following criteria:

- The glasses should exhibit the highest possible sulfate capacity without exceeding the critical [SO_4_^2−^] ion concentration, beyond which the precipitation of a highly soluble sulfate-bearing crystalline phase occurs (as observed in the XS_6PM system at a nominal 5 mol.% sulfate loading [80,81]), leading to its immediate release into the environment when the material is used in its intended applications;
- The glasses should contain comparable concentrations of sulfate ions;
- The P_2_O_5_ content is one of the key factors influencing the chemical reactivity of similar materials [59,74,103,127].

Considering these criteria, along with the analogous behavior observed for all [6P] glasses in the ‘in vitro’ experiment, the glasses 3S_6PM and 3S_10PM—both containing MgO as an alkaline earth metal modifier—were selected for further study. This selection enables the investigation of phosphorus content effects on dissolution kinetics while minimizing potential influences arising from variations in the characteristics of network-modifying cations on the release rate of glass components into the solution.

The shape of the corrosion kinetic curves obtained for both analyzed systems reflects two well-known kinetic regimes in glass dissolution studies in aqueous solutions [57,128,129], as shown in Figure 11a,b. The initial, highly intensive release of mobile species (K^+^, Mg^2+^) is associated with interdiffusion, where these cations undergo ion exchange with H^+^/H_3_O^+^ from the contacting solution, leaving the near-surface region of the glass depleted of these elements. The recorded release profiles of the network modifiers follow a t 12 dependence, a clear indicator of a transport-controlled process [130,131,132]. Surprisingly, larger PO_4_^3−^ and SO_4_^2−^ ions, which do not participate in proton exchange, are released following the same trend. Their initially rapid leaching can be attributed to their structural role in the glass matrix, where they exist as isolated units rather than being integrated into the silicate-based network; hence, their dissolution does not require the hydrolysis of silicon-oxygen bonds [59,133].

After approximately three hours of the initial rate regime, a noticeable decline (rate drop) appeared on the dissolution kinetic curves of the above-mentioned elements. Beyond this point, and continuing until the end of the experiment, a stable but nonzero residual release rate was observed (Figure 11a,b).

**Figure 12:**
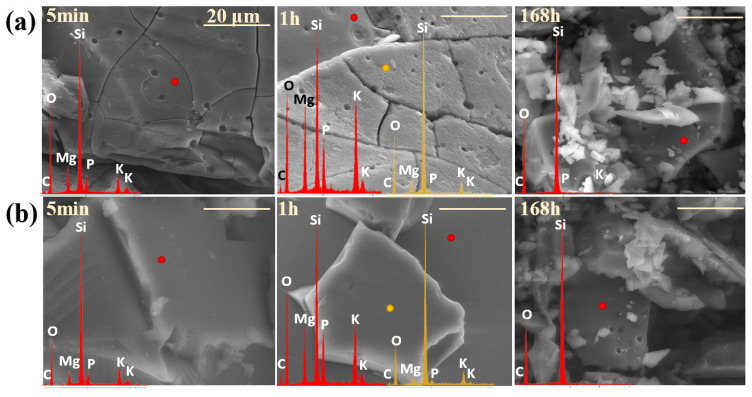
Micrographs and corresponding EDS spectra of 3S_6PM (**a**) and 3S_10PM (**b**) samples at different stages of long-term dissolution in 2% citric acid under dynamic leaching conditions: 5 min, 1 h, and 168 h. The micrographs were taken at 10,000× magnification, with the scale bar corresponding to 20 µm. The colors of the EDS spectra (yellow and red) correspond to the marked points in the respective micrographs, where the analyses were performed.

The observed rate drop in glass corrosion kinetics is typically attributed to two main phenomena: the formation of a passivating silica gel layer on the outermost glass surface in contact with the solution (1), or a decrease in reaction affinity due to saturation effects, where high concentrations of dissolved species in the leachate reduce the thermodynamic driving force for further dissolution (2) [57,134,135]. According to Equations (4) and (5) [136,137], hydration of the dealkalized glass surface layer is a natural consequence of the ion exchange process (as evidenced by the Si-rich layer depleted of mobile components, observed in micrographs of both glasses after 1h of long-term dissolution experiment; Figure 12a,b: yellow spectra, compared to the red ones, which represent initial compositions of the freshly exposed glass surfaces) and may lead to the reorganization of newly formed Si-OH groups into a more condensed, porous layer (6) and (7). However, even if such a passivating layer were to form, the ongoing disintegration of glass fragments (and the delaminating nature of its corrosion process, observable in Figure 11 and Figure 12 at the 1 h stage for both glasses) would continuously remove this surface area, exposing deeper regions of the glass and renewing the release of its components.

(4) $$\equiv Si-O^{-}-M_{\left(glass\right)}^{+}+H_{\left(sol.\right)}^{+}\to \equiv Si-{OH}_{\left(glass\right)}+M_{\left(sol.\right)}^{+}\left(\equiv represents\;the\;silica\;bonds\right)$$

(5) $$\equiv Si-O-M_{\left(glass\right)}^{+}+H_{3}O_{\left(sol.\right)}^{+}\to \equiv Si-{OH}_{\left(glass\right)}+M_{\left(sol.\right)}^{+}+H_{2}O$$

(6) $${\equiv Si-O-Si\equiv }_{\left(glass\right)}+{\mathrm{OH}}_{\left(sol.\right)}^{-}⇌{\equiv Si-OH}_{\left(glass\right)}+\equiv Si-O_{\left(glass\right)}^{-}$$

(7) $${\equiv Si-OH}_{\left(glass\right)}+\mathrm{HO}-{\mathrm{Si}\equiv }_{\left(glass\right)}⇌{\equiv Si-O-Si\equiv }_{\left(glass\right)}+H_{2}O$$

**Figure 13:**
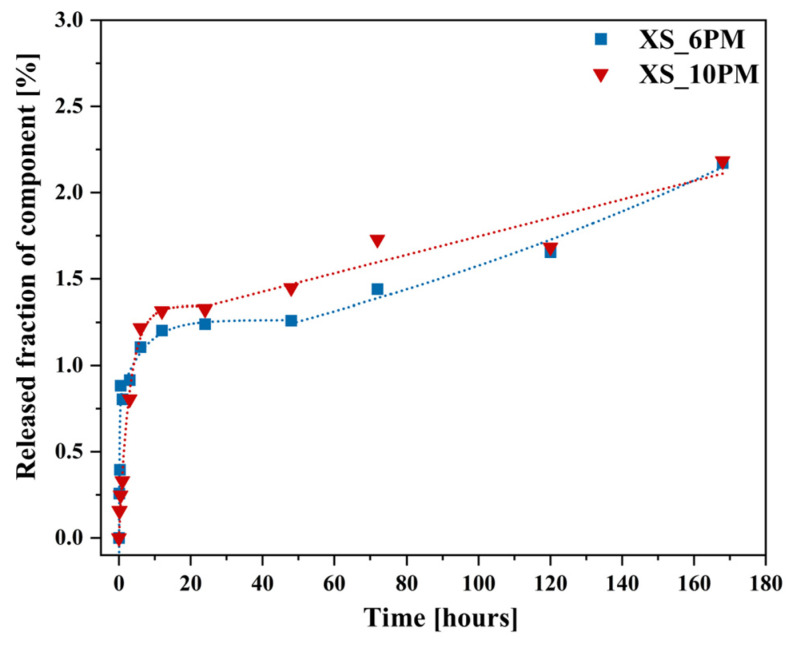
SiO_2_ dissolution profiles of 3S_6PM and 3S_10PM glasses in 2% citric acid under dynamic leaching conditions as a function of immersion time.

Hence, a hypothesis based on affinity effects appears more plausible, as the observed rate drop coincided with the depletion of a significant portion of mobile components from both glasses (~90% K^+^ and ~95% Mg^2+^). Nonetheless, the experimental conditions must be considered—specifically, the high dilution used in the dissolution tests, which suggests that saturation of the leachant with mobile species is unlikely [132]. Instead, the authors propose an alternative explanation for the observed rate drop under the given conditions: the open, fragmented structure of the designed materials facilitated the rapid leaching of mobile species from the most accessible regions of the glass via preferential ion transport pathways. The remaining, unreleased fraction of these elements was confined within less water-accessible regions of the glass network. Their liberation required the hydrolysis of strong ionic-covalent Si-O-Si bonds, which became the rate-limiting step in this kinetic regime. This conclusion is supported by the SiO_2_ release kinetics curves (Figure 13).

**Figure 14:**
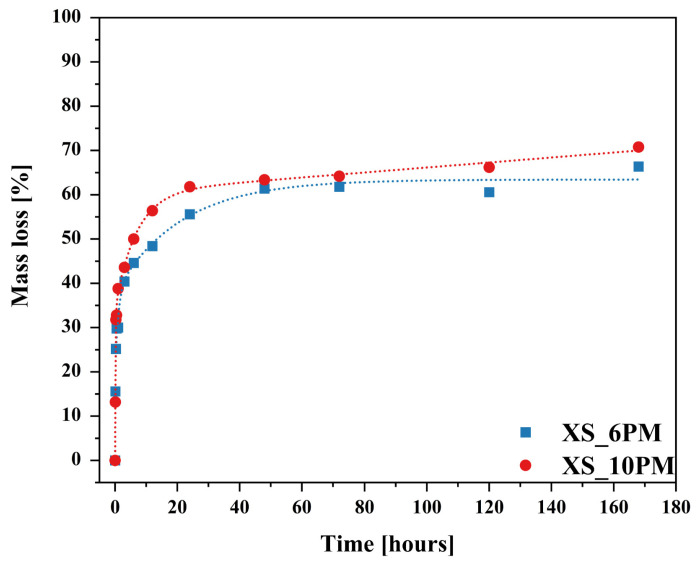
Mass loss curves of 3S_6PM and 3S_10PM glasses under dynamic leaching in a citric acid solution as a function of immersion time.

The dissolution profiles of silicon from both investigated glasses, despite an initial sharp release episode—likely attributed to the leaching of surface-exposed, loosely bound, or less network-integrated silica species [138,139]—did not follow the trends observed for the other glass constituents. After six hours of the experiment, a rate drop was observed; however, instead of stabilizing, the kinetic curve exhibited a gradual upward trend, increasing almost linearly with time, albeit at a slower rate than in the initial regime (Figure 13). According to [130,131,132], such a linear t^1^ dependence is indicative of a network hydrolysis process controlled by an interfacial reaction. The slowdown in the release of mobile glass components coincides with the moment when the rate of proton–metal exchange reactions decreases the overall glass dissolution rate, with kinetics now limited by the dissolution of the hydrated Si-O-Si framework [140]. Further insights can be drawn from the mass loss profiles of the studied glass samples throughout the experiment (Figure 14).

The curves presented in Figure 14 exhibit a similar trend to the release profiles of mobile components (Figure 11), with the key difference being that the slowdown occurred after 12 h rather than 6, as observed for the latter. Additionally, SEM micrographs displayed in Figure 11, taken at various exposure times to the aggressive solution, reveal a progressive exfoliation of surface fragments over time, accompanied by a noticeable reduction in the size of leached particles. These findings suggest that the delamination of the outermost glass layers—where surface dealkalization results in the formation of a mechanically weaker, silica-rich layer prone to detachment [140,141,142]—gradually exposed deeper yet intact regions of the glass, thereby sustaining the leaching of network-forming components and contributing to continuous mass loss.

**Figure 15:**
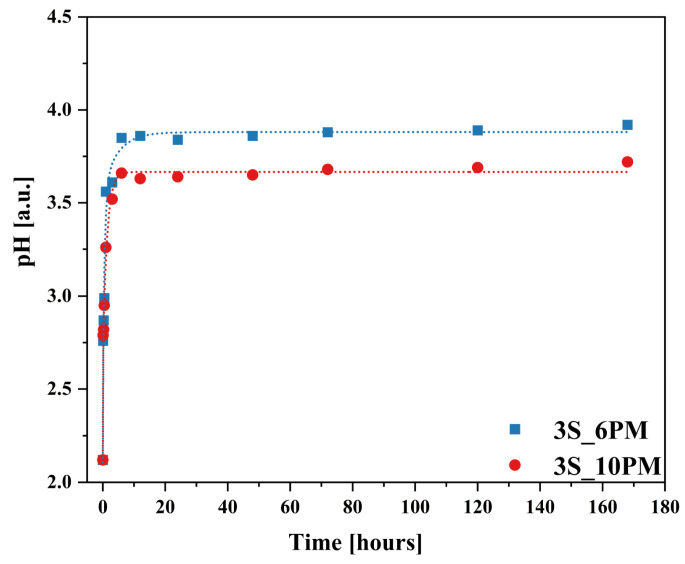
pH-change profiles of 3S_6PM and 3S_10PM glasses under dynamic leaching in citric acid as a function of immersion time.

It is also important to note the changes in the pH of the leachate as the leaching process progressed for both glass compositions (Figure 15), which closely corresponded with the results obtained by ICP-OES, showing a similar upward trend following the square root of time function (Figure 11).

The sharp increase in the release of mobile components into the solution during the first 6 h of the experiment (initial rate regime) coincided with a sudden rise in pH observed within this time range. After this period, once pH values reached 3.85 (for the XS_6PM system) and 3.66 (for the XS_10PM system), pH changed only slightly, ultimately stabilizing at 3.92 and 3.72, respectively (Figure 15). This observed increase in pH is a direct consequence of the interdiffusion process—proton consumption from the contact solution during the glass dealkalization led to the formation of hydroxyl groups in the leachate, subsequently raising its pH. The rate of glass transformation is therefore dependent on the concentration of available protons in the contacting solution. Although the pH variation profiles over the course of the experiment were similar for both systems, the samples representing the [10P] series showed a slightly smaller increase in pH compared to the other tested composition (Figure 15). This difference can be directly attributed to the higher content of orthophosphate [PO_4_^3−^] species in these glasses (see Section 2.1.3), whose mildly acidic nature may counterbalance the alkaline environment induced by the release of alkali or alkaline earth ions [43,143].

**Figure 16:**
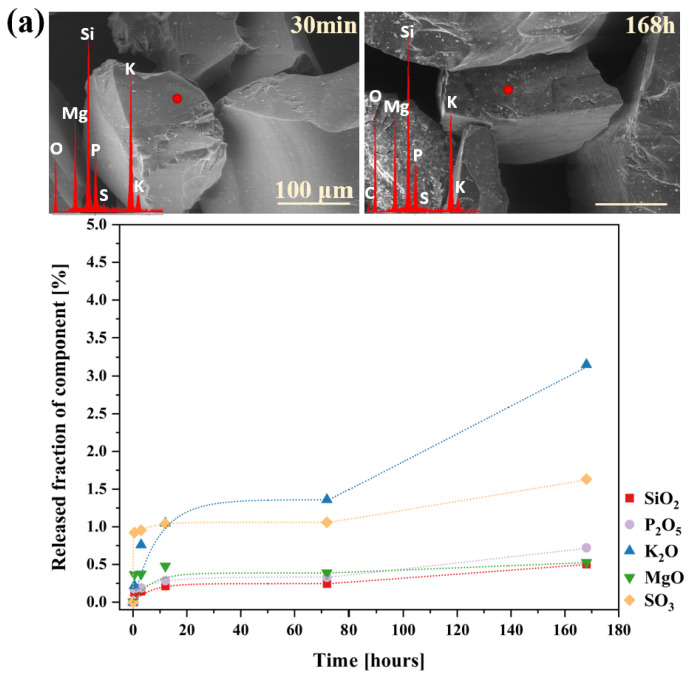

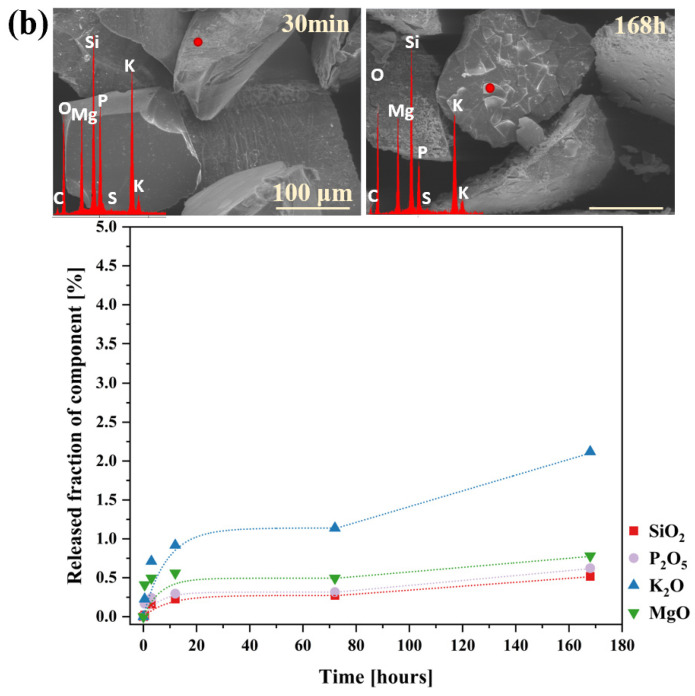
Ion dissolution profiles of 3S_6PM (**a**) and 3S_10PM (**b**) glasses in distilled water under dynamic leaching conditions as a function of immersion time, accompanied by micrographs and EDS data from selected surface points, illustrating surface changes at various stages of the experiment. The absence of [SO_4_^2−^] ion release profiles in the case of the 3S_10PM sample was due to the concentrations of this component in its leachate being below the sulfur detection limit of the ICP-OES method. The micrographs were taken at 1000× magnification, with the scale bar corresponding to 100 µm.

**Figure 17:**
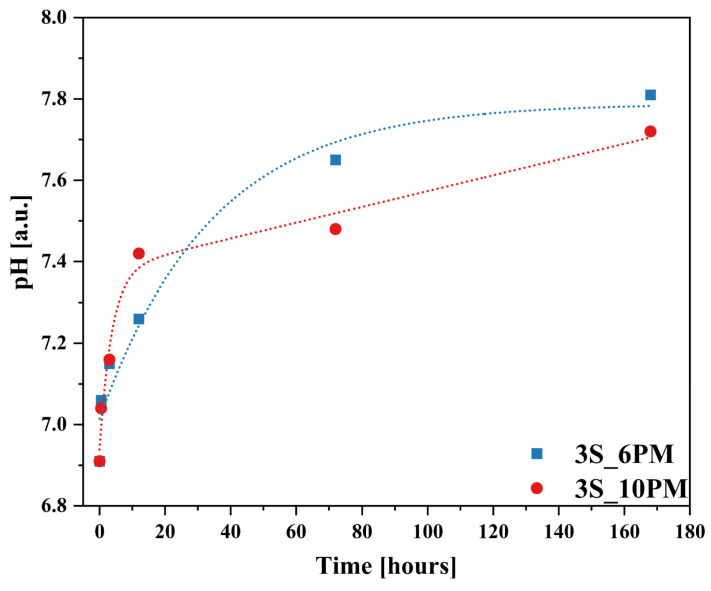
pH-change profiles of 3S_6PM and 3S_10PM glasses under dynamic leaching in distilled water as a function of immersion time.

Additionally, to characterize the behavior of the designed materials in a neutral medium, an analogous experiment was conducted using distilled water, with the results presented in Figure 16a,b and Figure 17. Interestingly, the obtained ion release profiles in the surrounding solution resembled those recorded for Si rather than for the mobile network-forming components. After an initial, more rapid release regime lasting approximately 3 h, which indicates the leaching of surface-exposed, loosely bound ions into the solution, the release rate slowed down, followed by a subsequent increase at 72 h (Figure 16a,b).

It is well established that an alkaline medium promotes network dissolution, in which OH^−^ ions attack the silicate network. During this process, new hydroxyl ions are generated, making the hydrolysis of the Si-O-Si glass backbone self-perpetuating, thus leading to the release of increasing amounts of glass components into the surrounding solution. However, it should be noted that these processes are most intense at pH values around 9 or higher [36,91,92,93], so the observed pH increase (Figure 17) in the case of the studied glasses is unlikely to be the cause of the renewed release after 72 h. Instead, the observed effect is more likely due to the previously mentioned exfoliation mechanism—peeling of hydrated surface flakes—which exposed previously unaffected areas of the glass, thereby further enhancing the release of the weakest, surface-bound components (Figure 16a,b: a slight decrease in K^+^ concentration was noticeable when comparing the EDS spectra of glasses after 30 min and 168 h).

It should be emphasized here that none of the components of the tested materials were leached into the surrounding solution in amounts exceeding 3.5% of their initial content in the glass. This finding highlights the significant resistance of the synthesized samples to the action of water.

Furthermore, following the approach used by Labbilta et al. [9,144,145], the initial dissolution rates (V_0_) of the studied glasses were calculated using the slope of the linear fit (V_0_ = dm/dt) from the mass loss dissolution curves (Figure 14). For comparison, the V_0_ values obtained for Si release in citric acid solution are also displayed in Table 2.

The data presented in Table 2 clearly demonstrate that the chemical resistance of the glasses was influenced not only by the characteristics of the corrosive medium but also by the composition and structure of the glass itself. Significantly higher values of initial dissolution rates (V_0_) observed for the citric acid solution, compared to those for Si release from the glasses, support the theory of an open, fragmented silica-oxygen network in the tested materials. This structure facilitated the leaching of ions localized within its voids while undergoing only limited hydrolysis, as confirmed by the negligible V_0_ values derived from the Si release curves. The elevated V_0_ values obtained in the acidic medium are primarily attributed to intense ion exchange processes within the pH range it represents, in contrast to distilled water, where only surface dealkalization occurs in the case of the tested samples. Furthermore, the higher initial dissolution rates observed for the [10P] sample, in both acidic and aqueous environments, coupled with the lower V_0_ for Si release, confirm a greater chemical reactivity, resulting from the presence of a larger number of independent [PO_4_^3−^] units prone to leaching, and simultaneously, an enhanced degree of polymerization of its silicon-oxygen subnetwork, as indicated by MAS-NMR studies.

It is important to emphasize that the data presented in this subsection reflect ion leaching profiles under dynamic conditions in highly acidic solutions, which, while accelerating ion exchange processes, do not directly replicate natural soil environments [140,146]. Instead, they illustrate general trends in the behavior of the studied materials. Additionally, these data were obtained without accounting for the complexities of the final application environment, such as the presence of exogenous elements, seasonal temperature fluctuations, or variations in water availability, all of which can influence ion transport. To address these factors, future studies will extend this investigation by evaluating material performance and assessing ion release dynamics under conditions that more closely mimic natural soil environments.

## 3. Conclusions

The structure of glass is inherently not in thermodynamic equilibrium, making it susceptible to chemical reactions that can lead to degradation over time. While this characteristic is often regarded as a major drawback, in the case of materials designed as vitreous carriers of plant-essential ions, it is precisely what enables their functionality. Such materials should release the elements encapsulated within their vitreous matrix in response to plant demand—triggered by organic acids secreted by root systems or soil microorganisms. This controlled release minimizes the risk of over-fertilization and, consequently, nutrient runoff, which could otherwise disrupt the biogeochemical equilibrium of the soil environment. Thus, this study aimed to design and synthesize vitreous carriers of micro- and macroelements, with a particular focus on sulfur-containing compositions, as potential fertilizers of a slow-release mechanism.

This study has demonstrated that the melt fritting method successfully yields amorphous materials, with only the XS_6PC and XS_10PM samples exhibiting subtle reflections indicative of the presence of stable phases, likely double potassium-calcium and potassium-magnesium phosphates, respectively. Notably, the tendency of these systems to reorganize was amplified by sulfate incorporation, which—by occupying voids in the matrix—induced structural stresses that lowered the energy barrier for crystallization.

It was confirmed that the alteration behavior of the tested glasses can be tailored at the compositional level. Even a slight variation in the type of alkaline earth network modifier was shown to influence the material’s structure and, consequently, its ion-release profile. MAS-NMR analysis revealed that all synthesized glasses (41SiO_2_–6(10)P_2_O_5_–20K_2_O–33(29)MgO/CaO/MgO + CaO) exhibited an open network, primarily composed of $Q_{\mathrm{Si}}^{2}$ silicate tetrahedra (short chains or rings), with minor contributions from $Q_{\mathrm{Si}}^{3}$ species and, in higher P_2_O_5_-content formulations, traces of $Q_{\mathrm{Si}}^{1}$/$Q_{\mathrm{Si}}^{4}$ units, whereas the phosphorus-oxygen subnetwork was exclusively composed of isolated $Q_{P}^{0}$ phosphate species. An experiment simulating exposure to acidic exudates from plant roots and microorganisms confirmed that this fragmented network facilitates ion exchange, with Si–O–Si hydrolysis occurring only at later stages of glass–solution interactions. Additionally, the incorporation of sulfur subtly modulated network cross-linking via the K^+^-scavenging effect of [SO_4_^2−^] species, which enhanced polymerization of the silicate backbone, which was further demonstrated via the ‘in vitro’ experiment, where this increased network connectivity translated into a reduced tendency to release mobile components into the solution.

Remarkedly, the conducted experiment also revealed that glass reactivity is not solely dictated by Q^n^ species distribution or framework stability but is strongly influenced by the surrounding environment—despite high solubility in acidic conditions, all tested glasses exhibited significant resistance to distilled water, as evidenced by the minimal leaching of matrix components.

A correlation between thermal behavior and chemical reactivity was also established: the presence of CaO, acting exclusively as a network modifier, led to the formation of the most fragmented and open network, reflected in the lowest thermal stability of the Si–O–Si backbone and its pronounced tendency to crystallize into more stable phases. This low stability and high network openness resulted in the highest SiO_2_ release, serving as an indicator of overall glass degradation. Conversely, MgO, acting both as a network modifier and a former present in the XS_6PM system, increased network polymerization, enhancing silicate backbone rigidity and reducing Si release. Meanwhile, the co-addition of MgO and CaO seemed to induce a mixed alkaline earth effect, known to increase the activation energy for ionic diffusion, as the respective samples exhibited the lowest glass transition temperature and chemical reactivity in terms of silica dissolution among the [6P] series. Given the highest NC of all the studied formulations, unsurprisingly, the XS_10P samples released SiO_2_ in the lowest amounts, despite their lowest thermal stability, stemming from the high concentration of incompatible [SiO_4_^4−^, PO_4_^3−^] and [SO_4_^2−^] species within the common framework. Additionally, the incorporation of sulfate units was evidenced to indirectly impact the behavior of the tested materials in the solutions used—the resulting increase in the network connectivity and rigidity and simultaneous decrease in thermal stability diminished their susceptibility to dissolution in acidic environments.

Meanwhile, dissolution kinetics studies provided insights into nutrient release profiles, which are essential for ensuring a steady supply of nutrients to plants: after an initial rapid dissolution rate in citric acid (strongly intensified by higher P_2_O_5_ content), the release of beneficial elements stabilized and became dependent on the hydrolysis of the silicate backbone. Importantly, the dissolution curves followed a t^1^/^2^ dependency (indicative of diffusion-controlled ion exchange), indicating that the process is governed by interdiffusion reactions. Furthermore, preliminary observations suggest that these materials underwent surface cracking, flaking, and exfoliation, which further enhanced ion leaching by increasing the contact area with the surrounding solution.

The present study employed laboratory experiments to gain a deeper understanding of the composition–structure–property relationship in sulfate-bearing multicomponent silicate-phosphate glasses—an essential foundation for future investigations under soil-conditions. The findings confirm that the synthesized glasses meet all the key requirements for potential glass fertilizers. Not only did they exhibit an amorphous nature, but they also possessed an open, fragmented network that facilitated water infiltration. Moreover, the designed materials demonstrated high resistance to water while selectively releasing their constituents in acidic solutions that simulated root exudates.

Overall, these findings establish a foundation for the rational design of sulfate-bearing silicate-phosphate glasses with controlled chemical activity, and—recognizing that laboratory data may not fully capture the complexities of the application environment—will pave the way for further studies aimed at evaluating their long-term behavior under realistic soil conditions.

## 4. Materials and Methods

### 4.1. Glass Design, Synthesis, and Preliminary Characterization (XRF, XRD, DSC, MAS-NMR)

In order to design vitreous carriers for macro- and micronutrients displaying prolonged release characteristics, raw materials were selected as sources of phosphorus ((NH_4_)_3_PO_4_), potassium (K_2_CO_3_), magnesium (MgO), calcium (CaO), and sulfur (K_2_SO_4_). Additionally, silica (SiO_2_) was chosen as the primary structural component of the glass matrix, serving as a matrix for these elements. To assess the feasibility of controlling the release of nutrients into the soil environment through the appropriate selection of glass matrix components, four formulations were developed based on previous experimental findings [3,16,80,81,96], which exhibited the highest potential for application as sulfate-bearing vitreous carriers of beneficial nutrients. The molar percentages of individual oxides in each formulation were as follows (X represents the molar percentage of SO_3_ introduced into the formulations; in this study, X = 0, 1, 3, or 5 mol.%):

- XS_6PM system: 41SiO_2_∙6P_2_O_5_∙20K_2_O∙33MgO∙XSO_3_
- XS_6PC system: 41SiO_2_∙6P_2_O_5_∙20K_2_O∙33CaO∙XSO_3_
- XS_6PMC system: 41SiO_2_∙6P_2_O_5_∙20K_2_O∙16.5MgO∙16.5CaO∙XSO_3_
- XS_10PM system: 41SiO_2_∙10P_2_O_5_∙20K_2_O∙29MgO∙XSO_3_

**Figure 18:**
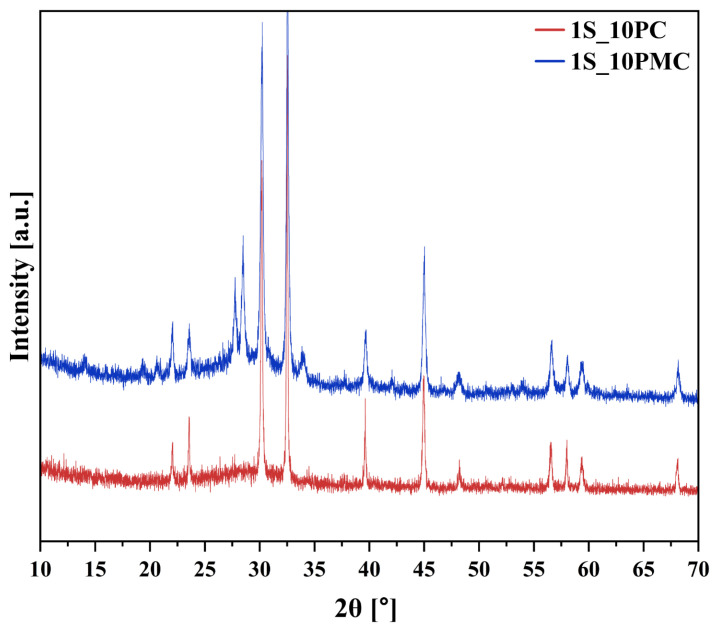
Diffraction patterns of 1S_10PC and 1S_10PMC samples.

It is important to emphasize that samples representing the XS_10PC and XS_10PMC compositions were also prepared to explore a selected range of possible combinations within the chosen P_2_O_5_ content regimes. However, the resulting materials exhibited the highest fraction of crystalline phases in their amorphous matrices, rendering them unsuitable for further testing (Figure 18).

The designed glasses were synthesized on a laboratory scale (~100 g) by melting precisely weighed (to an accuracy of 0.001 g) and homogenized (using a mortar and pestle) raw materials of analytical grade purity (SiO_2_, (NH_4_)_2_HPO_4_, K_2_CO_3_, MgO, and K_2_SO_4_, all supplied by CHEMPUR, Piekary Śląskie, Poland). The synthesis was carried out in ceramic crucibles placed in an electric chamber furnace under an air atmosphere at 1450 °C. The resulting melts were rapidly quenched by pouring them into cold water to obtain fully amorphous materials and to simultaneously suppress spontaneous crystallization [81]. The actual oxide compositions of the synthesized glasses were verified using X-ray fluorescence spectrometry (XRF) with an ARL Perform’X spectrometer, at the accredited testing laboratory FerroCarbo (Krakow, Poland). Samples for measurement were prepared from homogenized glass material in the form of either pressed or fused pellets (~10 g of glass). Potential losses associated with the high-temperature synthesis of the glasses were accounted for in the calculations, resulting in a measured composition that exhibited a high degree of consistency with the nominal values. The fusion method involved pre-calcination and melting of the samples in a fluxer.

Notably, the total oxide contents determined for the sulfur-containing silicate-phosphate glasses representing the XS_6PM and XS_10PM systems did not sum to 100%. This discrepancy likely resulted from the volatility of certain glass components during the fusion process at 1100 °C, which occurred as part of the XRF pearl preparation method.

The XRF-determined molar contents of individual glass components are presented in Table 3.

**Table 3:** Measured compositions of the synthesized specimens. Registered minor discrepancies between the expected and actual amounts of glass components are attributed to inherent measurement uncertainties and potential volatilization during the heating process.

No	SiO_2_	P_2_O_5_	K_2_O	MgO	CaO	SO_3_
0S_6PM	41.42 (±0.40)	6.75 (±0.09)	20.42 (±0.00)	30.17 (±0.45)	−	−
1S_6PM	37.39 (±0.41)	5.96 (±0.09)	19.53 (±0.00)	31.72 (±46)	−	0.84 (±0.12)
3S_6PM	40.00 (±0.41)	6.92 (±0.09)	20.63 (±0.00)	32.67 (±0.46)	−	1.84 (±0.14)
5S_6PM	41.60 (±0.40)	6.49 (±0.40)	17.69 (±0.00)	33.44 (±0.40)	−	1.59 (±0.40)
0S_6PC	39.11 (±0.44)	6.29 (±0.09)	18.88 (±0.00)	−	33.63 (±0.71)	−
1S_6PC	38.28 (±0.45)	6.14 (±0.10)	17.21 (±0.00)	−	35.79 (±0.73)	1.00 (±0.16)
3S_6PC	37.90 (±0.45)	6.46 (±0.09)	18.40 (±0.00)	−	35.12 (±0.72)	2.05 (±0.13)
5S_6PC	43.25 (±0.45)	6.10 (±0.01)	15.03 (±0.00)	−	37.42 (±0.72)	1.39 (±0.15)
0S_6PMC	39.91 (±0.41)	6.33 (±0.09)	18.40 (±0.00)	15.46 (±0.34)	18.16 (±0.44)	−
1S_6PMC	38.20 (±0.42)	6.33 (±0.09)	19.59 (±0.00)	15.53 (±0.33)	18.09 (±0.56)	0.95 (±0.12)
3S_6PMC	38.08 (±0.09)	6.64 (±0.09)	18.92 (±0.00)	16.57 (±0.34)	20.15 (±0.45)	1.76 (±0.15)
5S_6PMC	41.70 (±0.42)	6.46 (±0.09)	16.41 (±0.00)	16.32 (±0.35)	20.89 (±0.56)	1.40 (±0.14)
0S_10PM	38.11 (±0.40)	11.01 (±0.08)	21.56 (±0.00)	29.12 (±0.44)	−	−
1S_10PM	39.00 (±0.42)	10.89 (±0.09)	19.95 (±0.00)	28.98 (±0.50)	−	0.25 (±0.05)
3S_10PM	39.37 (±0.42)	11.02 (±0.09)	21.14 (±0.00)	29.35 (±0.47)	−	0.83 (±0.10)
5S_10PM	42.29 (±0.41)	11.48 (±0.08)	22.29 (±0.00)	24.71 (±0.46)	−	0.48 (±0.07)

To verify the amorphous nature of the synthesized materials, the samples were ground into a powder form (<0.1 mm) and analyzed using X-ray diffraction (XRD) on a PANalytical X’Pert Pro diffractometer equipped with a Cu anode (Malvern, UK). Data were collected over the 2θ range of 10–70° with a goniometer step size of 0.008° and an angular reproducibility of ±0.0002°. Crystalline phases, if present, were identified using the HighScore Plus software (https://www.malvernpanalytical.com/en/products/category/software/x-ray-diffraction-software/highscore-with-plus-option, accessed on 2 April 2025).

Thermal property variations as a function of sulfate content were examined using a STA 449 F3 Jupiter (NETZSCH, Selb, Germany) instrument operating in heat-flux DSC mode, with a temperature resolution of 0.001 K, at the Thermophysical Research Laboratory of the Faculty of Materials Science and Ceramics, AGH University of Krakow. To conduct differential scanning calorimetry (DSC) measurements, powder samples (31 mg) with grain sizes ranging from 0.1 to 0.3 mm were placed in platinum crucibles and heated at a rate of 10 °C min^−1^ in a dynamic air atmosphere (80 mL min^−1^). The instrument temperature sensor was calibrated using the melting temperatures of high-purity reference materials (Al, Zn, Sn, Au, Ag), and sensitivity for enthalpy and heat capacity determinations was calibrated with synthetic sapphire (α-Al_2_O_3_) standard references. The Netzsch Proteus Thermal Analysis Program (version 5.0.0) was used to determine the characteristic glass transition temperature (T_g_), the onset (T_x_), and the maximum (T_c_) of the glass crystallization effect. T_g_ was determined as the midpoint of the relevant transformation step, while T_x_ and T_c_ were derived from the onset and maximum of the exothermic crystallization peak, respectively.

High-resolution solid-state ^29^Si and ^31^P MAS-NMR spectra were recorded using an APOLLO console (Tecmag, Houston, TX, USA) and a 7 T/89 mm superconducting magnet (Magnex, Oxford, UK) at the Jerzy Haber Institute of Catalysis and Surface Chemistry, Polish Academy of Sciences. A Bruker HP-WB high-speed MAS probe, equipped with a 4 mm zirconia rotor and KEL-F cap, was employed for sample spinning. A single 3 µs RF pulse, corresponding to a π/2 flipping angle, was applied. The ^31^P MAS-NMR spectra were measured at a resonance frequency of 121.264 MHz, with the sample spinning at 6 kHz. The initial ^31^P T1 relaxation time was estimated to be approximately 3 s. The acquisition delay was set to 10 s, with 256 scans acquired. The ppm frequency scale was referenced to the ^31^P resonance of 85% H_3_PO_4_. For the ^29^Si MAS-NMR spectra, measurements were taken at a resonance frequency of 59.515 MHz, with the sample spinning at 4 kHz. The initial ^29^Si T1 relaxation time was estimated to be approximately 10 s, with an acquisition delay of 30 s and 256 scans. The ppm frequency scale was referenced to the ^29^Si resonance of TMS (Tetramethylsilane). For the ^31^P MAS-NMR spectra, the uncertainties in line positions and linewidths were about 0.2 ppm, while the relative intensities had an accuracy of approximately 1%. For the ^29^Si spectra, the corresponding uncertainties were 0.4 ppm and 1%, respectively.

### 4.2. Glass Dissolution Studies (‘In Vitro’ Conditions)

The assessment of behavior of the designed materials in a solution simulating plant root exudates (hereafter referred to as the ‘in vitro’ experiment) was conducted in accordance with Regulation (EC) 2003/2003 on fertilizers, which provides guidelines for Member States regarding sampling and analytical methods for fertilizers [126]. Glass fractions of 0.1–0.3 mm were carefully weighed (to an accuracy of 0.001 g) to obtain 1 g specimens, which were placed in glass beakers. To each beaker, 100 (±1) mL of 2% citric acid solution was added, a common root exudate suitable for simulating plant action. The beakers were then covered with a watch glass and placed on magnetic stirrers, where they were mixed for 30 min at 300 rpm at room temperature. After this period, the contents of the beakers were immediately filtered into a dry container through a dry, phosphate-free filter paper. The collected filtrates were analyzed for pH using a Mettler Toledo pH meter and subsequently subjected to analysis via inductively coupled plasma optical emission spectroscopy (ICP-OES, PerkinElmer Optima 2000 DV, Norwalk, CA, USA) at the Accredited Hydrogeochemical Laboratory of AGH University of Krakow (Certificate No. AB 1050). The same procedure was applied to assess the solubility of the synthesized samples in distilled water, although this test was conducted only on selected samples representing all systems with 3 mol.% SO_3_ content.

The authors find it necessary to clarify the use of the term ‘in vitro’ in the context of this study, as it is traditionally associated with medical research. Notably, in soil science, specialists consider the soil environment a ‘living entity’ due to its complex biological activity and interactions with microorganisms. Based on this perspective, the term ‘in vitro’ refers here to laboratory-based experiments conducted outside soil system, such as dissolution studies of glass fertilizers in simulated environments (e.g., solutions that mimic soil conditions). However, to underscore the distinction from traditional biological applications, this term is presented in apostrophes throughout the manuscript.

### 4.3. Experimental Approach to Glass Dissolution Kinetics

The dissolution kinetics of selected glasses in a 2% citric acid solution was investigated for two compositions—3S_6PM and 3S_10PM. The experiment commenced by weighing glass fractions (0.1–0.3 mm) to a precision of 0.001 g, resulting in 1 g specimens of each composition. Twelve replicate samples (12 × 1 g) were prepared for each composition, placed in glass beakers, and 100 (±1) mL of 2% citric acid solution was added to each. The beakers were then covered with watch glasses and placed on magnetic stirrers, where they were stirred for 30 min at 300 rpm and room temperature. At intervals of 5, 15, and 30 min and 1 h, 3 h, 6 h, 12 h, 24 h, 48 h, 72 h, 120 h, and 168 h, leachates were collected by filtration through dry, phosphate-free corrugated filter paper, followed by pH measurement and composition analysis using ICP-OES. An analogous experiment was conducted to estimate the dissolution kinetics in distilled water, but with a lower frequency of sampling, at 30 min, 3 h, 12 h, 72 h, and 168 h. Additionally, selected residues left on the filter (after 5, 30, and 60 min and 6 h, 12 h, and 168 h of dissolution in citric acid, and after 30 min and 168 h in distilled water) were carefully cleaned by ultrasonic treatment to remove any remnants of corrosive solution. The residues were then weighed to a precision of 0.001 g and analyzed using Scanning Electron Microscopy (SEM) with Energy Dispersive X-ray Spectroscopy (EDS) for qualitative and semi-quantitative analyses at selected points on the sample surfaces. Observations were conducted under high-vacuum conditions using a backscatter electron detector (BSE) with an accelerated voltage of 18 kV employing a NOVA NANO SEM 200 (FEI EUROPE, Eindhoven, The Netherlands) coupled with an EDAX EDS analyzer in the Laboratory of Scanning Microscopy and Microanalysis at the Faculty of Materials Science and Ceramics, AGH University of Krakow.

## Figures and Tables

**Table 1:** DSC-registered parameters (T_g_, T_x_, and Angell parameter values) for all tested samples across the studied compositions.

No	Thermal Parameter		
	T_g_	T_x_	Angell (K_A_)
	[°C]		[a.u.]
0S_6PM	613	750	137.0
1S_6PM	615	741	126.2
3S_6PM	624	689	65.1
5S_6PM	636	687	50.8
0S_6PC	595	624	28.9
1S_6PC	594	615	21.1
3S_6PC	608	635	26.5
5S_6PC	626	663	36.6
0S_6PMC	550	586	36.1
1S_6PMC	564	594	29.5
3S_6PMC	584	613	29.4
5S_6PMC	589	625	36.3
0S_10PM	544	556	12.1
1S_10PM	544	552	7.3
3S_10PM	546	573	27.0
5S_10PM	549	601	51.6

**Table 2:** Initial dissolution rates (V_0_) of the 3S_6PM and 3S_10PM glasses, determined from the mass loss curves registered during leaching studies in citric acid and distilled water. For comparison, the V_0_ values corresponding to SiO_2_ release in citric acid solution are also included.

	Glass	
	3S_6PM	3S_10PM
	$V_{0}$ [g/h]	
Citric acid, 2%	0.432	0.576
Distilled water	0.018	0.024
Si initial release rate (citric acid)	0.006	0.001

## Data Availability

The data presented in this study are available on request from the corresponding author.
